# Additional Burden Averted in the United States From Use of MF59-Adjuvanted Seasonal Influenza Vaccine Compared With Standard Seasonal Influenza Vaccine Among Adults ≥65 Years

**DOI:** 10.1093/ofid/ofad429

**Published:** 2023-08-09

**Authors:** Ian McGovern, Aditya Sardesai, Alexandra Taylor, Hector Toro-Diaz, Mendel Haag

**Affiliations:** Seqirus USA Inc, Center for Outcomes Research and Epidemiology, Cambridge, Massachusetts, USA; Evidera, Evidence Synthesis, Modeling & Communication, San Francisco, California, USA; Evidera, Evidence Synthesis, Modeling & Communication, San Francisco, California, USA; Evidera, Evidence Synthesis, Modeling & Communication, Bethesda, Maryland, USA; Seqirus Netherlands BV, Center for Outcomes Research and Epidemiology, Amsterdam, The Netherlands

**Keywords:** disease burden, influenza, MF59-adjuvanted trivalent inactivated influenza vaccine, modeling, older adults

## Abstract

**Background:**

The MF59-adjuvanted trivalent inactivated influenza vaccine (aIIV3) is designed to overcome immunosenescence and enhance vaccine responses in older adults. We expanded on the Centers for Disease Control and Prevention (CDC) modeling method to estimate the number of additional influenza-related outcomes averted with aIIV3 versus generic quadrivalent inactivated influenza vaccine (IIV4) in adults ≥65 years over 3 influenza seasons (2017–2018 to 2019–2020) in the United States.

**Methods:**

A static compartmental model was developed based on an existing CDC model with 2 previously recommended calculation methods that increased the accuracy of the model in providing estimates of burden averted. Model inputs included vaccine effectiveness, vaccine coverage, population counts, and disease burden estimates. Additional burden averted (symptomatic cases, outpatient visits, hospitalizations, intensive care unit [ICU] admissions, and deaths) was expressed as total incremental cases averted between the vaccines. Sensitivity analyses tested the resilience of the model results to uncertainties in model inputs.

**Results:**

The model estimated that vaccination with aIIV3 versus IIV4 would avert 2.24 times as many symptomatic cases, outpatient visits, hospitalizations, ICU stays, and deaths during 2017–2018; the burden averted in 2018–2019 and 2019–2020 with aIIV3 would be 3.44 and 1.72 times that averted with IIV4, respectively. Disease burden estimates and relative vaccine effectiveness of aIIV3 had the greatest impact on model estimates.

**Conclusions:**

Over 3 influenza seasons, the model estimated that aIIV3 was more effective than IIV4 in averting influenza-related outcomes, preventing 1.72 to 3.44 times as many influenza illnesses with proportionate decreases in related healthcare use and complications.

## Teaser

A modeling analysis showed that MF59-adjuvanted trivalent inactivated influenza vaccine averted 1.72 to 3.44 times as many symptomatic cases and complications as a generic quadrivalent vaccine over 3 influenza seasons in adults ≥65 years of age.

Seasonal influenza poses a substantial public health burden on the general population, with the disease burden varying considerably among seasons. The Centers for Disease Control and Prevention (CDC) estimated that between 2010 and 2020, influenza resulted in 9–41 million illnesses, 140 000–710 000 hospitalizations, and 12 000–52 000 deaths annually [1]. Serious medical complications leading to hospitalizations and deaths disproportionately affect individuals aged ≥65 years [2, 3]. To mitigate the public health impact of influenza, routine annual vaccination is recommended for individuals 6 months and older [4]. However, because of age-related immunosenescence, standard-dose inactivated influenza vaccines may elicit a diminished immune response in older adults compared with younger age groups [5, 6]. To overcome this challenge, an MF59-adjuvanted vaccine and a higher-dose nonadjuvanted vaccine have been developed to enhance protection against influenza in older adults [7].

The trivalent formulation of the MF59-adjuvanted inactivated influenza vaccine (aIIV3) (FLUAD, Seqirus Inc) was first approved for use among adults ≥65 years of age in the United States (US) in 2015, and the quadrivalent formulation (aIIV4) was later approved in 2020 and was first available in the 2020–2021 season. A systematic review and meta-analysis of 21 noninterventional studies conducted during the 2006–2007 to 2019–2020 seasons showed that aIIV3 improved relative vaccine effectiveness (rVE) compared with IIV4 in adults ≥65 years [8].

Because of antigenic drift, which leads to the emergence of new strains, influenza vaccines must be reformulated every season. The degree of antigenic dissimilarity between circulating and vaccine strains and the predominance of those strains influence vaccine effectiveness. From 2004 to 2022, influenza vaccine effectiveness relative to no vaccination (absolute vaccine effectiveness [aVE]) has ranged between 12% and 50% among adults ≥65 years of age [9]. Similarly, the rVE of one vaccine compared with another often varies from one season to another and therefore must be regularly evaluated.

The impact of influenza vaccination is assessed annually because of seasonal variation in the epidemiologic characteristics of the season and variations in vaccine performance. Since 2010, the CDC has used monthly data on disease risk, vaccination coverage, and vaccine effectiveness to develop a national model to project the healthcare burden averted by vaccination compared with a “no vaccination” scenario [10, 11]. The CDC model had a simple structure that assumed a constant transmission of disease independent of case density and allowed direct use of CDC surveillance data, but it was subject to inaccuracies. To provide more accurate modeling methods, a subsequent study developed a static compartmental model that analyzed simulated daily counts of vaccinations and influenza cases to estimate the total burden averted by vaccination [12]. This model was considered the reference model, against which 7 alternative simpler static model methods based on monthly aggregated data were tested for accuracy. Two methods produced results closest to those of the reference model and were expected to improve the accuracy of the calculations of averted cases for future influenza seasons.

In this analysis, we used these 2 best-performing models to compare the estimated influenza-associated burden averted by vaccination with aIIV3 versus IIV4 in individuals aged ≥65 years over 3 influenza seasons.

## METHODS

### Model Design and Structure

The model was designed as a static compartmental model and was used to estimate the influenza burden averted during a given influenza season (defined as the interval from October to September of the following year). Compartmental models are a general modeling technique that assigns subjects to different “compartments” or health states (eg, susceptible or infected). Subjects can then transition between compartments over time (with each timepoint being 1 “cycle”) based on different model assumptions/input parameters. This model was based on a monthly cycle length and involved a pairwise comparison of 2 distinct vaccination scenarios. Influenza burden estimates were based on data reported by the CDC during the 2017–2018, 2018–2019, and 2019–2020 influenza seasons. Burden averted (symptomatic cases, outpatient visits, hospitalizations, intensive care unit [ICU] admissions, and deaths) was expressed as the total cases prevented for each vaccine as well as the incremental cases prevented between the vaccines (ie, total averted aIIV3 − total averted IIV4).

The design of the model was based on 2 previously recommended calculation methods to estimate disease burden averted by vaccinations [12]. These 2 methods (referred to as Method 2 and Method 3 as per Tokars et al [12], both based on a monthly cycle) had performed well against a reference model (based on a daily cycle) when tested for accuracy of the results across several influenza seasons. The reference model included 7 health states that were defined through combinations of patient status variables (ill [case] or well [non-case], vaccinated or nonvaccinated, and immune or susceptible) (Supplementary Figure 1). For both models, each of the input parameters were used to calculate the number of subjects in each of the 7 health states (ie, “compartments”) for each month, and then the cumulative number of outcomes that occurred was calculated and subtracted from the number of outcomes expected if none of the subjects were vaccinated.

The model structure for Method 2 was similar to that of the reference model but did not include any immunity lag. Furthermore, Method 2 applied vaccine coverage and effectiveness only to the noncase population rather than to the total population as in the reference model. The model structure in Method 3 also did not include an immunity lag but calculated the proportion infected and vaccinated in each cycle by applying the current-month case and vaccination counts to the prior-month compartment values, as opposed to the reference model, which calculated all states concurrently. Analyses using both Methods 2 and 3 showed a minimal difference (<1%) between the methods in symptomatic cases averted when aIIV3 was compared with IIV4 over the 2019–2020 influenza season in adults aged ≥65 years (Supplementary Table 1). Method 2 was therefore selected for the base-case analysis of aIIV3 and IIV4 because of its simpler and more transparent structure. All models were implemented in Microsoft Excel software.

### Model Inputs

Vaccine effectiveness, vaccine coverage, population counts, and disease burden estimates were obtained from the literature and CDC surveillance data. Data for the 2017–2018, 2018–2019, and 2019–2020 influenza seasons were included in the model.

#### Population Size

The population size of 52 788 000 adults ≥65 years of age was based on 2019 US census data [13] and was assumed to be constant across seasons.

#### Vaccine Effectiveness

Vaccine effectiveness, the percentage of patients who achieved immunity after vaccination, was defined with absolute or relative estimates. Based on the inputs available in the literature, aVE was derived for both vaccines. Overall (any vaccine) aVE values published by the CDC for the ≥65 years age group for prevention of outpatient visits were used as a proxy for aVE of IIV4. Overall aVE values published by the CDC for the age group ≥65 years were 17%, 12%, and 37% for the 2017–2018, 2018–2019, and 2019–2020 seasons, respectively [9]. The aVE of aIIV3 was estimated by back-calculating the aVE based on the assumed aVE of IIV4 and previously published season-specific estimates of the rVE of aIIV3 versus IIV4. The rVE estimates for aIIV3 versus IIV4 for prevention of influenza-related medical encounters (ie, outpatient/emergency room visits or hospitalizations) were sourced from published studies that used the same database and analysis methods, to improve comparability across the 3 seasons. Estimates used for the 2017–2018, 2018–2019, and 2019–2020 seasons were 20.8% [14], 26.0% [14], and 27.5% [15], respectively. The aVE of aIIV3 was calculated using the following equation [16]:


$$aVE_{aIIV3}=[rVE_{aIIV3\;vs\;IIV4}\;\times (1-aVE_{IIV4})+aVE_{IIV4}].$$

#### Vaccine Coverage

Vaccine coverage was defined as the percentage of the total ≥65 years age group population vaccinated in each season. Seasonal monthly vaccine coverage values were extracted from the CDC Weekly Influenza Vaccination Dashboard [17]. To align the CDC vaccine coverage for each season (defined as July to May of the following year) with the season definition in the current model (October to September), the model added vaccination coverage from July to September to the count for October and assumed that coverage in each of these months was the same as that reported for May. Annual vaccine coverage rates for adults ≥65 years for the 2017–2018, 2018–2019, and 2019–2020 seasons were 60%, 68%, and 70%, respectively [18]. Monthly vaccine coverage rates for the ≥65 years age group for each season are shown in Supplementary Table 2.

#### Incidence Timing and Monthly Case Distribution

**Figure 1.**
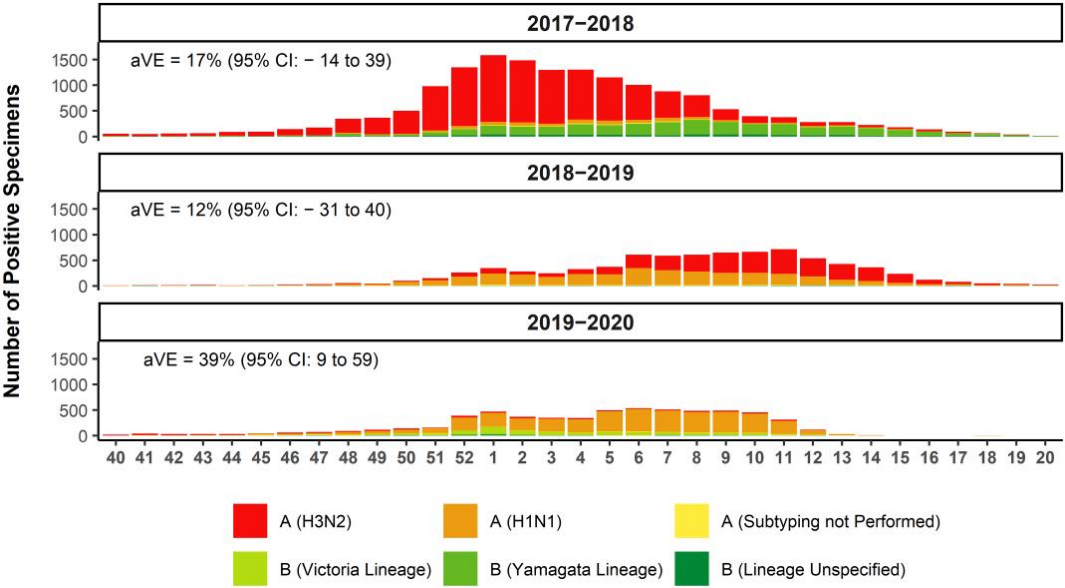
Weekly influenza-positive specimens by strain over 3 influenza seasons in adults aged ≥65 years reported by public health laboratories in the United States to the Centers for Disease Control and Prevention. Abbreviations: aVE, absolute vaccine effectiveness; CI, confidence interval.

The monthly case distribution used in the model was derived from the seasonal weekly number of positive influenza specimens for all strains (H1N1, H3N2, B/Victoria, B/Yamagata, and unsubtyped influenza A and B) based on influenza subtyping performed by sentinel public health laboratories and published by the CDC [19]. The weekly distribution of influenza strains over the 2017–2018, 2018–2019, and 2019–2020 seasons in individuals ≥65 years is shown in Figure 1. To obtain monthly case counts, weekly counts were mapped to the corresponding month each year. The distribution of cases each month was then calculated as the percentage of cases in a month out of the total cases for the season. Monthly case distributions over the 3 influenza seasons are listed in Supplementary Table 3.

#### Estimates of Disease Burden of Influenza

**Figure 2.**
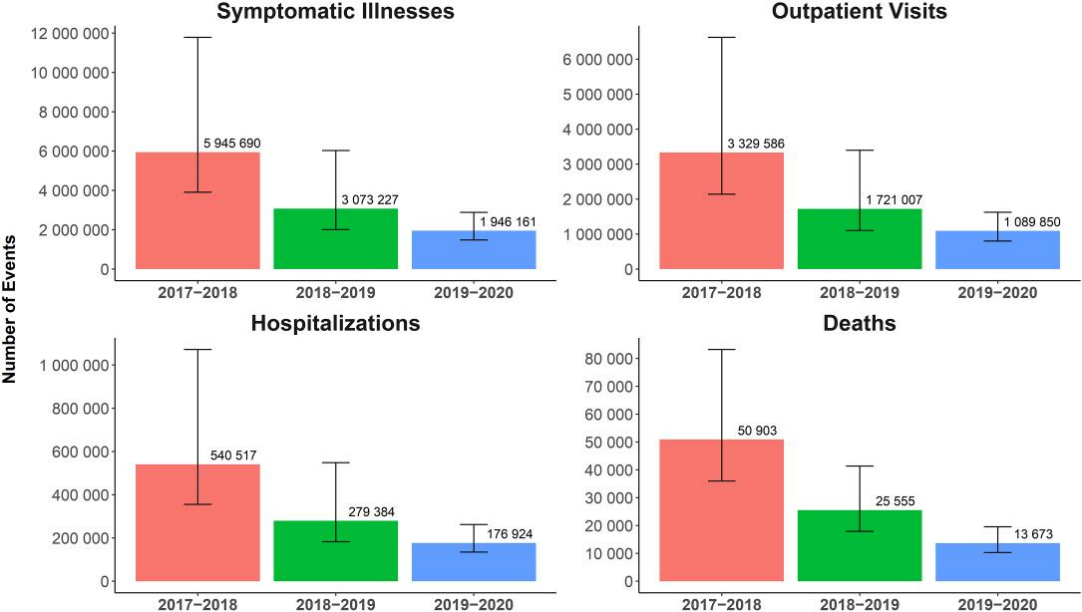
Estimated number of influenza-related events over 3 influenza seasons in adults aged ≥65 years. Data are presented with point estimate and 95% credible interval.

Disease burden was estimated as the number of symptomatic cases, outpatient visits, hospitalizations, ICU visits, and deaths related to influenza. Burden estimates were based on data on hospitalizations obtained from the Influenza Hospitalization Surveillance Network (FluSurv-NET), which conducts population-based surveillance for laboratory-confirmed, influenza-associated hospitalizations [20]. Specifically, the CDC estimates influenza incidence in specific US hospitals and then calculates the number of influenza cases and influenza-related outpatient visits using a fixed ratio relative to the total hospitalizations [21]. Influenza-related deaths were estimated based on the ratio of deaths to hospitalizations in a given season [21]. Influenza-related ICU admissions were estimated as a proportion of hospitalizations because an ICU visit was defined as a hospitalization that required admission to the ICU. ICU admission rates for the 2017–2018 and 2018–2019 seasons were available from the CDC; the ICU rate for the 2019–2020 season was calculated as the average of ICU rates from the 2010–2011 season to the 2018–2019 season [20]. CDC-estimated incidence rates of influenza-related symptomatic illnesses, outpatient visits, hospitalizations, and deaths for adults ≥65 years for the 3 influenza seasons are shown in Figure 2. ICU admission rate estimates for the 2017–2018, 2018–2019, and 2019–2020 seasons were 15.5%, 17.8%, and 17.4%, respectively.

### Sensitivity Analyses

Deterministic sensitivity analyses (DSAs) and probabilistic sensitivity analyses (PSAs) were performed to evaluate the impact of uncertain model input parameters on model outcomes. DSAs used fixed upper and lower bounds (eg, 95% confidence intervals [CIs]) to evaluate how results change at the top and bottom of expected ranges. When available, the lower and upper bounds used in the DSA were sourced from the 95% CIs reported in the literature or by the CDC. When no CIs were reported, the bounds were assumed to be ±20% around the point estimate used in the base case. Results of DSAs corresponding to the lower and upper bounds of the tested parameters were ranked based on their absolute difference and presented as a tornado diagram for the incremental outcome averted for the ≥65 years age group. The point estimates and ranges for each parameter varied in the DSA for the 3 influenza seasons are listed in Supplementary Table 4.

PSAs were also performed to evaluate the impact of parameter uncertainty by repeatedly sampling from a specified distribution of input parameters. For each comparison, 1000 PSA simulations were run to generate the empirical distribution for burden averted. Results of PSAs consistently stabilized before 1000 simulations. Beta distributions were assumed for aVE of IIV4, rVE of aIIV3, and ICU admission rate; for all other parameters, a normal distribution was assumed. The impact of uncertainty in multiple parameters was evaluated simultaneously, and results were presented as incremental cases averted along with quartiles. Standard error values of the PSA parameters are shown in Supplementary Table 5.

## RESULTS

### Burden Averted With aIIV3 Versus IIV4 in Adults Aged ≥65 Years

**Figure 3.**
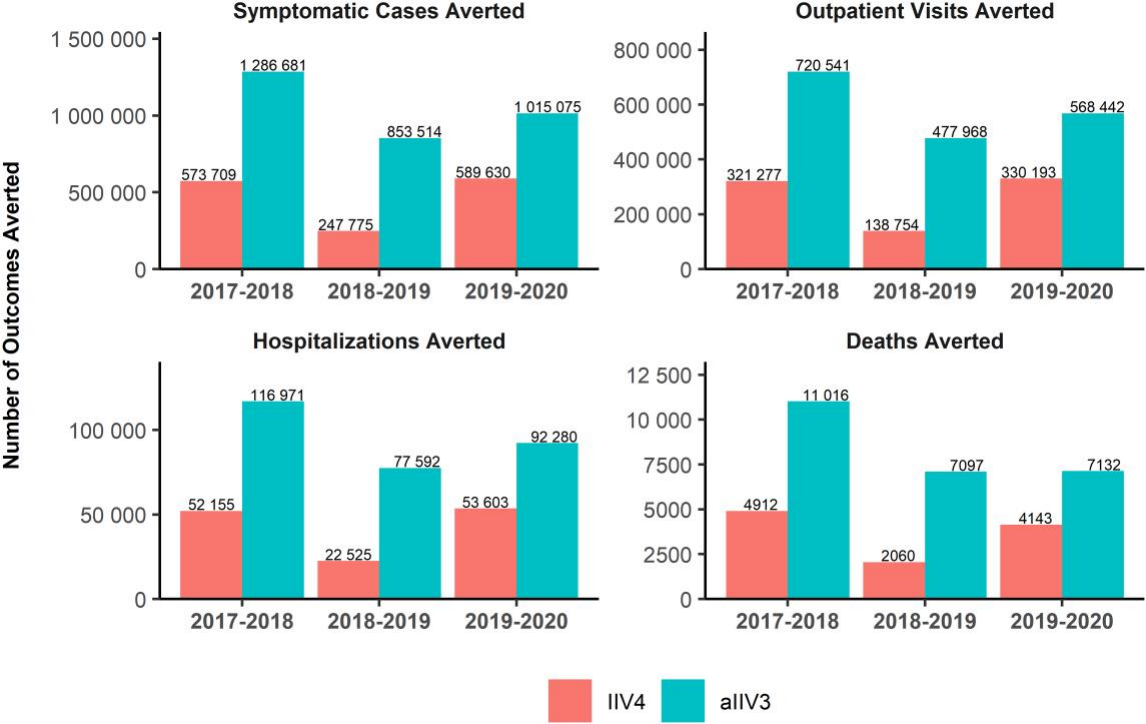
Model-estimated outcomes averted by vaccination with MF59-adjuvanted trivalent inactivated influenza vaccine (aIIV3) versus generic quadrivalent inactivated influenza vaccine (IIV4) over 3 influenza seasons in adults aged ≥65 years.

In individuals aged ≥65 years, vaccination with aIIV3 lowered the disease burden when compared with IIV4 in all 3 seasons. During the 2017–2018 season, use of aIIV3 by all vaccinated adults ≥65 years would have prevented 1 286 681 of the 5 945 690 symptomatic illnesses that occurred in that age group (including vaccinated and unvaccinated individuals), compared with 573 709 illnesses prevented if everyone had received IIV4, an increase of 712 972 illnesses prevented by aIIV3 (Table 1 and Figure 3). Use of aIIV3 would also have prevented an additional 399 261 outpatient visits, 64 816 hospitalizations, 10 046 ICU admissions, and 6104 deaths. For each of the outcomes, use of aIIV3 would have prevented 2.24 times as many influenza-related events compared with IIV4. For the other 2 seasons, use of aIIV3 compared with IIV4 would have prevented 3.44 times as many influenza-related events in 2018–2019 and 1.72 times as many events in 2019–2020. The number of additional influenza-related events prevented was highest for the 2017–2018 season because of the high incidence of influenza that season.

**Table 1.** Influenza-Related Burden Averted With MF59-Adjuvanted Trivalent Inactivated Influenza Vaccine (IIV) Versus Generic Quadrivalent IIV Over 3 Influenza Seasons in Adults ≥65 Years

Season	aVE, IIV4	rVE, aIIV3 vs IIV4	Vaccine Coverage	Outcome	Total Events	Events Prevented, aIIV3	Events Prevented, IIV4	Additional Events Prevented, aIIV3 vs IIV4
2017–2018	17%	20.8%	60%	Symptomatic cases	5 945 690	1 286 681	573 709	712 972
				Outpatient visits	3 329 586	720 541	321 277	399 261
				Hospitalizations	540 517	116 971	52 155	64 816
				ICU admissions	81 618	18 130	8084	10 046
				Deaths	50 903	11 016	4912	6104
2018–2019	12%	26.0%	68%	Symptomatic cases	3 073 227	853 514	247 775	605 739
				Outpatient visits	1 721 007	477 968	138 754	339 214
				Hospitalizations	279 384	77 592	22 525	55 067
				ICU admissions	49 730	13 811	4009	9802
				Deaths	25 555	7097	2060	5037
2019–2020	37%	27.5%	70%	Symptomatic cases	1 946 161	1 015 075	589 630	425 445
				Outpatient visits	1 089 850	568 442	330 193	238 249
				Hospitalizations	176 924	92 280	53 603	38 677
				ICU admissions	30 608	15 937	9257	6679
				Deaths	13 673	7132	4143	2989

Abbreviations: aIIV3, MF59-adjuvanted trivalent inactivated influenza vaccine; aVE, absolute vaccine effectiveness; ICU, intensive care unit; IIV4, generic quadrivalent inactivated influenza vaccine; rVE, relative vaccine effectiveness.

The model results showed that the number needed to vaccinate (NNV) to avoid any influenza-related outcome was lower for aIIV3 compared with IIV4 in all seasons. In the 2017–2018 season, aIIV3 more than halved the NNV to prevent a single symptomatic case (from 54 with IIV4 to 24 with aIIV3) (Supplementary Table 6). Since NNV is inversely related to burden averted, the NNV was largest for less frequent outcomes (eg, hospitalizations and deaths) and smallest for more frequent outcomes (eg, symptomatic illness and outpatient visits), but in all cases was lower with aIIV3 than with IIV4. Use of aIIV3 would result in the greatest reduction in NNV to prevent a single symptomatic case during the 2018–2019 season (from 144 with IIV4 to 42 with aIIV3), showing that improved vaccine effectiveness is likely to have the greatest proportionate impact (but not necessarily overall impact) on prevention of influenza-related events during seasons with low absolute vaccine effectiveness (aVE of IIV4 during the 2018–2019 season was only 12%).

### Deterministic Sensitivity Analysis

**Figure 4.**
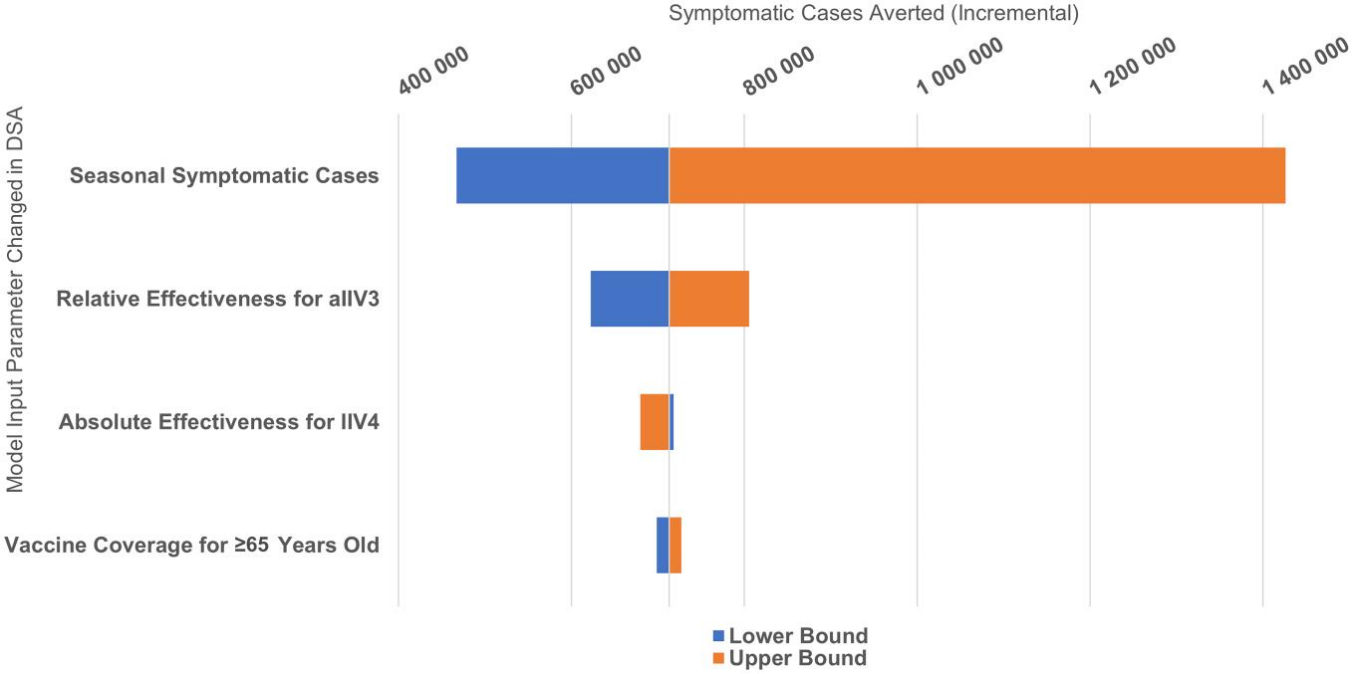
Tornado diagram of additional (MF59-adjuvanted trivalent inactivated influenza vaccine vs generic quadrivalent inactivated influenza vaccine) symptomatic cases averted in adults aged ≥65 years for the 2017–2018 influenza season. Lower bound and upper bound represent the use lower/upper limits of the 95% confidence intervals of the estimates for the specified model input variables. Abbreviations: aIIV3, MF59-adjuvanted trivalent inactivated influenza vaccine; DSA, deterministic sensitivity analysis; IIV4, generic quadrivalent inactivated influenza vaccine.

The parameters of interest evaluated in the DSA were aVE, rVE, vaccine coverage, and individual burden outcomes. The input parameter with the largest impact on the 2017–2018 season model results for any outcome was the incidence of the outcome of interest; other influential parameters were the rVE of aIIV3 versus IIV4 and vaccine coverage (Supplementary Table 7). DSA results for symptomatic cases averted with aIIV3 versus IIV4 in 2017–2018 are shown in Figure 4 and Supplementary Table 8. Results for the other outcomes in a given season followed the same trend because of the same underlying input parameters (other than burden counts), as shown by the results for the 2019–2020 influenza season for symptomatic cases, outpatient visits, hospitalizations, ICU visits, and deaths averted (Supplementary Figures 2–6, respectively). The aVE of IIV4 had a nonlinear influence on incremental outcomes. This finding was related to how the aVE of aIIV3 was calculated with respect to the IIV4 aVE. Although increasing absolute effectiveness of IIV4 increased the resulting aVE of aIIV3, the absolute difference between the 2 could decrease, resulting in a reduction in the additional outcomes averted. Across seasons, burden estimates were the most influential parameter, followed by rVE; vaccine coverage and aVE were the third or fourth most impactful, depending on the season (Figure 4 and Supplementary Figures 2–9).

Use of Method 2 as the base case over Method 3 was validated using the 2019–2020 season data, which showed there was no meaningful change in the DSA results for symptomatic cases averted and deaths averted, respectively, between Method 2 (Supplementary Figures 2 and 6) and Method 3 (Supplementary Figures 10 and 11).

### Probabilistic Sensitivity Analysis

Based on the results of 1000 simulations, the mean number of additional symptomatic cases averted with the use of aIIV3 over IIV4 during the 2017–2018 influenza season was 709 203 (interquartile range [IQR], 545 120–868 033) (Supplementary Figure 12), compared with 712 972 in the base-case deterministic analysis (Supplementary Table 8). The results of PSAs had comparable distributions for the other seasons and other outcomes (in terms of proportionate change from base-case results) (Supplementary Figures 13–20). In each of the seasons, the mean PSA results were within a 1% difference of the base case results and the IQRs were −23% to +22% of base case in 2017–2018, −23 to +21% in 2018–2019, and −14 to +12% in 2019–2020.

A comparison of Method 2 (Supplementary Figures 16 and 20) and Method 3 (Supplementary Figures 21 and 22) for the base-case scenario in 2019–2020 showed that the distributions of symptomatic cases and deaths were similar between the methods, with a slightly lower shift for the Method 3 distributions.

## DISCUSSION

In the US, the seasonal circulation of influenza viruses causes a substantial public health and economic burden. Serious medical complications leading to hospitalizations and deaths are typically greatest among individuals aged ≥65 years, as shown previously with influenza-attributed hospitalization rates [3]. The Advisory Committee on Immunization Practices (ACIP) recommends annual vaccination against influenza for all persons aged ≥6 months to reduce morbidity and mortality caused by influenza [22]. As of the 2022–2023 season, ACIP recommends that adults ≥65 years receive an adjuvanted or higher-dose or recombinant vaccine [23]. The CDC routinely publishes surveillance data and has also developed a model that estimates the total number of influenza illnesses, medical visits, and hospitalizations prevented by vaccination using season-specific data on burden of disease, vaccine coverage, and vaccine effectiveness [21]. This model was used as the basis of the current analysis. Although this analysis was conducted prior to the updated ACIP recommendation, these findings help to reinforce the recommendation by demonstrating the public health impact of the use of enhanced influenza vaccines instead of standard influenza vaccines.

During the 2017–2018 season, the rVE for aIIV3 versus IIV4 was 20.8%; as a result, aIIV3 prevented 2.24 times as many influenza infections and related complications as IIV4. The greatest proportionate increase in burden averted was during the 2018–2019 season because of the high rVE for aIIV3 (26.0%) and low aVE (12%), which resulted in a 3.44-fold increase in burden averted for aIIV3 versus IIV4. However, despite having the highest proportionate increase in burden averted, the 2018–2019 season had the lowest total burden averted because of the relatively low aVE that season. By contrast, the 2019–2020 season had the lowest proportionate increase in burden averted for aIIV3 (1.72 times as many events prevented) despite having the highest rVE of aIIV3 versus IIV4 (27.5%); this was due to the relatively high aVE (37%) for standard vaccines that season. If all vaccinated adults ≥65 years had received aIIV3, the vaccine would have prevented 22% of all cases (including unvaccinated cases) in the 2017–2018 season, 28% in 2018–2019, and 52% in 2019–2020. These results demonstrate that the public health impact of improved vaccine effectiveness is influenced by multiple factors and is not always intuitive if only rVE is considered.

Key drivers of variability in model results were the seasonal burden estimates reported by the CDC, which had wide 95% CIs, and the rVE of aIIV3. The likely explanation for the higher impact of rVE compared with aVE on model results is that the aVE of IIV4 also influenced the derived aVE of aIIV3 and thus mitigated its impact on incremental outcomes. The PSA data also suggested that despite uncertainty around parameters, the PSA results aligned with the base-case results showing that aIIV3 was associated with additional burden averted compared with IIV4, with the majority of simulation within a less than 25% change from baseline. To improve comparability across seasons, the studies selected for rVE estimates were conducted using the same database and population and comparable analysis methods.

The best available evidence was used to inform the model, but there were limitations that could influence model results. First, the model used a static structure that did not account for herd immunity and reduced transmission, so the model would likely underestimate the true cases averted. A dynamic model would be needed to account for herd immunity. Second, overall aVE estimates reported by the CDC were used as a proxy for aVE of IIV4. However, the estimate of the overall VE for IIV4 likely included people vaccinated with enhanced vaccines, particularly in later seasons, which would have improved the overall VE. Thus, the aVE used in the model for IIV4 is likely to be higher than that expected in regular clinical practice, resulting in an overestimation of cases averted by the generic vaccine. Third, it was assumed that vaccination could only prevent an outcome by preventing infection and did not include the possibility of reducing severity of disease among breakthrough vaccinated cases (eg, a patient only requiring outpatient care who may have been hospitalized if unvaccinated). Fourth, the disease burden estimates reported by the CDC correspond to the overall population, which included both vaccinated and nonvaccinated individuals, and were therefore an underestimation of the disease burden that would be observed in an entirely unvaccinated population. This would likely result in an underestimation of the true cases averted. Finally, age-specific data were not available for the ICU admission rate, and overall age group data were used for the rVE of aIIV3.

## CONCLUSIONS

In this analysis, a static compartmental model was used to estimate the burden of disease averted by influenza vaccination with aIIV3 versus IIV4 in individuals aged ≥65 years over 3 seasons in the US. The model estimated that vaccination with aIIV3 prevented 1.72 to 3.44 times as many symptomatic cases, outpatient visits, hospitalizations, ICU stays, and deaths compared with use of IIV4 over each season examined.

## Supplementary Material

ofad429_Supplementary_Data

## Supplementary Data

Supplementary materials are available at *Open Forum Infectious Diseases* online. Consisting of data provided by the authors to benefit the reader, the posted materials are not copyedited and are the sole responsibility of the authors, so questions or comments should be addressed to the corresponding author.

## Notes

***Author contributions.*** Conceptualization: I. M. Methodology: I. M., A. S., A. T., H. D. Software: A. S., A. T., H. D. Validation: I. M., A. S., A. T., H. D. Formal analysis: A. S., A. T., H. D. Investigation: I. M., A. S., A. T., H. D. Resources: M. H. Data curation: I. M., A. S., A. T., H. D. Writing—original draft preparation: I. M., M. H. Writing—review and editing: I. M., A. S., A. T., H. D., M. H. Visualization, I. M., A. S., A. T., H. D. Supervision: I. M., M. H. Project administration: I. M., A. S., A. T., H. D. Funding acquisition: M. H. All authors have read and agreed to the published version of the manuscript.

***Acknowledgments.*** Meher M. Dustoor, PhD, and Esther Tazartes, MS, of Global Outcomes Group provided editorial assistance; these services were funded by CSL Seqirus.

***Data availability.*** Model details and all model input data are publicly available from the sources indicated within the manuscript.

***Patient consent.*** This study does not include factors necessitating patient consent.

***Financial support.*** This study was supported by CSL Seqirus.
