# Supplementary material for: Additional Burden Averted in the United States From Use of MF59-Adjuvanted Seasonal Influenza Vaccine Compared With Standard Seasonal Influenza Vaccine Among Adults ≥65 Years
Source: Open Forum Infect Dis. 2023 Aug 9;10(8):ofad429. doi: 10.1093/ofid/ofad429 (PMC10438869; doi:10.1093/ofid/ofad429)

**Supplementary Table 1. Comparison of Method 2 and Method 3 for Burden Averted with aIIV3 versus IIV4 over the 2019-2020 Influenza Season in Adults ≥65 Years**

| **Method** | **Outcome** | **Events Prevented aIIV3** | **Events Prevented IIV4** | **Additional Events Prevented aIIV3 vs IIV4** |
| --- | --- | --- | --- | --- |
| 2 | Symptomatic cases | 1,015,075 | 589,630 | 425,445 |
|  | Outpatient visits | 568,442 | 330,193 | 238,249 |
|  | Hospitalizations | 92,280 | 53,603 | 38,677 |
|  | ICU admissions | 15,937 | 9257 | 6679 |
|  | Deaths | 7132 | 4143 | 2989 |
| 3 | Symptomatic cases | 1,014,348 | 589,331 | 425,017 |
|  | Outpatient visits | 568,035 | 330,026 | 238,010 |
|  | Hospitalizations | 92,214 | 53,576 | 38,638 |
|  | ICU admissions | 15,925 | 9253 | 6673 |
|  | Deaths | 7126 | 4140 | 2986 |

Abbreviations: aIIV3, MF59-adjuvanted trivalent inactivated influenza vaccine; ICU, intensive care unit; IIV4, generic quadrivalent inactivated influenza vaccine.

**Supplementary Table 2. Monthly Vaccine Coverage over the 2017-2018, 2018-2019, and 2019-2020 Influenza Seasons in Adults ≥65 Years**

| **Month** | **2017-2018** | **2018-2019** | **2019-2020** |
| --- | --- | --- | --- |
| October | 38% | 42% | 46% |
| November | 49% | 54% | 58% |
| December | 52% | 59% | 63% |
| January | 56% | 63% | 67% |
| February | 57% | 65% | 69% |
| March | 58% | 67% | 69% |
| April | 59% | 68% | 70% |
| May | 60% | 68% | 70% |
| June | 60% | 68% | 70% |
| July | 60% | 68% | 70% |
| August | 60% | 68% | 70% |
| September | 60% | 68% | 70% |

Source: Centers for Disease Control and Prevention. Influenza vaccination coverage for persons 6 months and older. 2021. Accessed March 30, 2023. <https://www.cdc.gov/flu/fluvaxview/interactive-general-population.htm>

**Supplementary Table 3. Monthly Distribution of Influenza Cases over the 2017-2018, 2018-2019, and 2019-2020 Influenza Seasons in Adults ≥65 Years**

| **Month** | **2017-2018** | **2018-2019** | **2019-2020** |
| --- | --- | --- | --- |
| October | 2% | 1% | 2% |
| November | 5% | 1% | 4% |
| December | 19% | 7% | 13% |
| January | 41% | 18% | 33% |
| February | 19% | 29% | 33% |
| March | 8% | 28% | 14% |
| April | 4% | 10% | 0% |
| May | 0% | 1% | 0% |
| June | 0% | 1% | 0% |
| July | 0% | 1% | 0% |
| August | 0% | 1% | 2% |
| September | 0% | 1% | 4% |

Based on data from the CDC. Source: Centers for Disease Control and Prevention. Age group distribution of influenza positive specimens reported by public health laboratories, national summary.. <https://gis.cdc.gov/grasp/fluview/flu_by_age_virus.html>

**Supplementary Table 4. DSA Parameter Values for 2017-2018, 2018-2019, and 2019-2020 Influenza Seasons in Adults ≥65 Years**

| **Parameter** | **Base Case** | **Lower Bound** | **Upper Bound** | **Source** |
| --- | --- | --- | --- | --- |
| **2017-2018** |  |  |  |  |
| aVE for IIV4 | 17% | 0%^a^ | 39% | CDC^1^ |
| rVE of aIIV3 | 20.8% | 18.4% | 23.2% | Boikos et al.^3^ |
| Symptomatic cases | 5,945,690 | 3,907,025 | 11,786,777 | CDC^2^ |
| Outpatient visits | 3,329,586 | 2,139,716 | 6,623,717 | CDC^2^ |
| Deaths | 50,903 | 35,989 | 83,230 | CDC^2^ |
| Hospitalizations | 540,517 | 355,184 | 1,071,525 | CDC^2^ |
| ICU admission rate | 15.5% | 12.4% | 18.6% | Assume ± 20% of base-case value |
| **2018-2019** |  |  |  |  |
| aVE of IIV4 | 12% | 0%^a^ | 40% | CDC^1^ |
| rVE of aIIV3 | 26.0% | 23.4% | 28.6% | Boikos et al.^3^ |
| Symptomatic cases | 3,073,227 | 2,008,898 | 6,030,701 | CDC^2^ |
| Outpatient visits | 1,721,007 | 1,097,482 | 3,394,980 | CDC^2^ |
| Deaths | 25,555 | 17,874 | 41,363 | CDC^2^ |
| Hospitalizations | 279,384 | 182,627 | 548,246 | CDC^2^ |
| ICU admission rate | 17.8% | 14.2% | 21.4% | Assume ±20% of base-case value |
| **2019-2020** |  |  |  |  |
| aVE of IIV4 | 37% | 5% | 58% | CDC^1^ |
| rVE of aIIV3 | 27.5% | 24.4% | 30.5% | Imran et al^4^ |
| Symptomatic cases | 1,946,161 | 1,480,075 | 2,883,659 | CDC^2^ |
| Outpatient visits | 1,089,850 | 797,945 | 1,621,738 | CDC^2^ |
| Deaths | 13,673 | 10,298 | 19,568 | CDC^2^ |
| Hospitalizations | 176924 | 134,552 | 262,151 | CDC^2^ |
| ICU admission rate | 17.4% | 13.8% | 20.7% | Assume ± 20% of base-case value |

Abbreviations: aIIV3, MF59-adjuvanted trivalent inactivated influenza vaccine; aVE, absolute vaccine effectiveness; DSA, deterministic sensitivity analysis; ICU, intensive care unit; IIV4, generic quadrivalent inactivated influenza vaccine; rVE, relative vaccine effectiveness.

^a^Reported value was negative; bound was set to zero %.

Sources:

1. Centers for Disease Control and Prevention. Past season’s vaccine effectiveness estimates. 2022. Accessed January 6, 2023. <https://www.cdc.gov/flu/vaccines-work/past-seasons-estimates.html>
2. Centers for Disease Control and Prevention.Centers for Disease Control and Prevention. 2022. Accessed January 6, 2023. https://www.cdc.gov/flu/about/burden/past-seasons.html
3. Boikos C, Fischer L, O'Brien D, et al. Relative effectiveness of adjuvanted trivalent inactivated influenza vaccine versus egg-derived quadrivalent inactivated influenza vaccines and high-dose trivalent influenza vaccine in preventing influenza-related medical encounters in US adults ≥ 65 years during the 2017-2018 and 2018-2019 influenza seasons. Clin Infect Dis **2021**;73(5):816-23.
4. Imran M, Puig-Barbera J, Ortiz JR, et al. Relative effectiveness of MF59 adjuvanted trivalent influenza vaccine vs nonadjuvanted vaccines during the 2019-2020 influenza season. Open Forum Infect Dis **2022**;9(5):ofac167.

**Supplementary Table 5. Standard Error Values for PSA Parameters for 2017-2018, 2018-2019, and 2019-2020 Influenza Seasons in Adults ≥65 Years**

| **Parameter** | **2017-2018** | **2018-2019** | **2019-2020** | **Distribution** | **Source** |
| --- | --- | --- | --- | --- | --- |
| aVE of IIV4 | 13.5% | 18.1% | 13.5% | Beta | CDC^1-3^ |
| rVE of aIIV3 | 1.2% | 1.3% | 1.6% | Beta | Boikos et al^4^  Imran et al^5^ |
| Symptomatic cases | 2,010,141 | 1,025,970 | 358,057 | Normal | CDC^6^ |
| Outpatient visits | 1,143,878 | 586,096 | 210,151 | Normal | CDC^6^ |
| Hospitalizations | 182,740 | 93,270 | 32,551 | Normal | CDC^6^ |
| Deaths | 12,051 | 5992 | 2365 | Normal | CDC^6^ |
| ICU admission rate | 1.6% | 1.8% | 1.7% | Beta | Assumption |

Abbreviations: aIIV3, MF59-adjuvanted trivalent inactivated influenza vaccine; aVE, absolute vaccine effectiveness; ICU, intensive care unit; IIV4, generic quadrivalent inactivated influenza vaccine; PSA, probabilistic sensitivity analysis; rVE, relative vaccine effectiveness.

Sources:

1. Centers for Disease Control and Prevention. Seasonal influenza vaccine effectiveness, 2017-2018. 2019. Accessed January 6, 2023. <https://www.cdc.gov/flu/vaccines-work/2017-2018.html>
2. Centers for Disease Control and Prevention. US Flu VE data for 2018-2019. 2019. Accessed January 6, 2023. <https://www.cdc.gov/flu/vaccines-work/2018-2019.html>
3. Centers for Disease Control and Prevention. US Flu VE data for 2019-2020. 2020. Accessed January 6, 2023. <https://www.cdc.gov/flu/vaccines-work/2019-2020.html>
4. Boikos C, Fischer L, O'Brien D, et al. Relative effectiveness of adjuvanted trivalent inactivated influenza vaccine versus egg-derived quadrivalent inactivated influenza vaccines and high-dose trivalent influenza vaccine in preventing influenza-related medical encounters in US adults ≥ 65 years during the 2017-2018 and 2018-2019 influenza seasons. Clin Infect Dis **2021**;73(5):816-23.
5. Imran M, Puig-Barbera J, Ortiz JR, et al. Relative effectiveness of MF59 adjuvanted trivalent influenza vaccine vs nonadjuvanted vaccines during the 2019-2020 influenza season. Open Forum Infect Dis **2022**;9(5):ofac167.
6. Centers for Disease Control and Prevention.Centers for Disease Control and Prevention. 2022. Accessed January 6, 2023. https://www.cdc.gov/flu/about/burden/past-seasons.html

**Supplementary Table 6. Number Needed to Vaccinate for aIIV3 Versus
IIV4 over the 2017-2018, 2018-2019, and 2019-2020 Influenza Seasons in Adults ≥65 Years**

| **Parameter** | **aIIV3** | **IIV4** | **Incremental** |
| --- | --- | --- | --- |
| **2017-2018** |  |  |  |
| Symptomatic cases | 24 | 54 | -30 |
| Outpatient visits | 43 | 96 | -53 |
| Hospitalizations | 265 | 594 | -329 |
| ICU admissions | 1708 | 3831 | -2123 |
| Deaths | 2812 | 6306 | -3494 |
| **2018-2019** |  |  |  |
| Symptomatic cases | 42 | 144 | -102 |
| Outpatient visits | 75 | 258 | -183 |
| Hospitalizations | 461 | 1588 | -1127 |
| ICU admissions | 2589 | 8919 | -6330 |
| Deaths | 5039 | 17,357 | -12,318 |
| **2019-2020** |  |  |  |
| Symptomatic cases | 36 | 62 | -26 |
| Outpatient visits | 65 | 111 | -47 |
| Hospitalizations | 398 | 685 | -287 |
| ICU admissions | 2303 | 3964 | -1662 |
| Deaths | 5146 | 8859 | -3713 |

Abbreviations: aIIV3, MF59-adjuvanted trivalent inactivated influenza vaccine; ICU, intensive care unit; IIV4, generic quadrivalent inactivated influenza vaccine.

**Supplementary Table 7. Input Parameter Ranking of Incremental Burden Averted Outcomes in Adults ≥65 Years (aIIV3 vs IIV4) for the 2017-2018 influenza Season (DSA)**

| **Rank** | **Symptomatic Cases** | **Deaths** |
| --- | --- | --- |
| Seasonal symptomatic cases | **1** | **5** |
| rVE of aIIV3 vs IIV4 | **2** | **2** |
| aVE of IIV4 | **3** | **3** |
| Vaccine coverage | **4** | **4** |
| Seasonal deaths | **-** | **1** |

The ranking indicates the order in which changes in the parameters influence the result of each model outcome. The parameter with the largest impact is represented in the darkest tone.

Abbreviations: aIIV3, MF59-adjuvanted trivalent inactivated influenza vaccine; aVE, absolute vaccine effectiveness; DSA, deterministic sensitivity analysis; IIV4, generic quadrivalent inactivated influenza vaccine; rVE, relative vaccine effectiveness.

**Supplementary Table 8. Top Input Parameters for Additional (aIIV3 vs IIV4) Symptomatic Cases Averted in Adults ≥65 Years for the 2017-2018 Influenza Season (DSA)**

| **Rank** | **Parameter Group** | **Lower Bound** | **Upper Bound** | **Lower Bound: % Change from Base Case** | **Upper Bound: % Change from Base Case** |
| --- | --- | --- | --- | --- | --- |
|  | Base case | 712,972 |  |  |  |
| 1 | Symptomatic cases | 467,148 | 1,426,031 | -34.5% | 100.0% |
| 2 | rVE for aIIV3 vs IIV4 | 622,587 | 805,751 | -12.7% | 13.0% |
| 3 | aVE for IIV4 | 717,941 | 679,805 | 0.7% | -4.7% |
| 4 | Vaccine coverage | 698,849 | 727,226 | -2.0% | 2.0% |

Abbreviations: aIIV3, MF59-adjuvanted trivalent inactivated influenza vaccine; aVE, absolute vaccine effectiveness; DSA, deterministic sensitivity analysis; IIV4, generic quadrivalent inactivated influenza vaccine; rVE, relative vaccine effectiveness.

**Supplementary Figure 1. Generalized Structure for the Reference Model.**


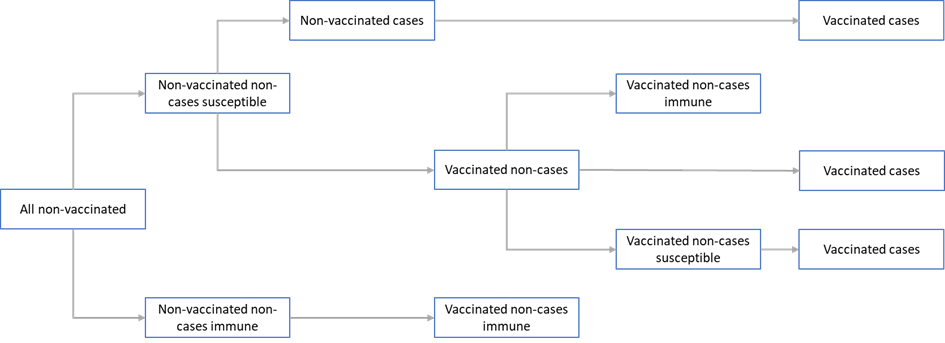


**General note for Supplementary Figures 2 to 22:**

Unless specifically mentioned, Method 2 was used for all analyses.

**Supplementary Figure 2. Tornado Diagram of Additional (aIIV3 vs IIV4) Symptomatic Cases Averted in Adults ≥65 Years for the 2019-2020 Influenza Season.** Abbreviations: aIIV3, MF59-adjuvanted trivalent inactivated influenza vaccine; IIV4, generic quadrivalent inactivated influenza vaccine.

**
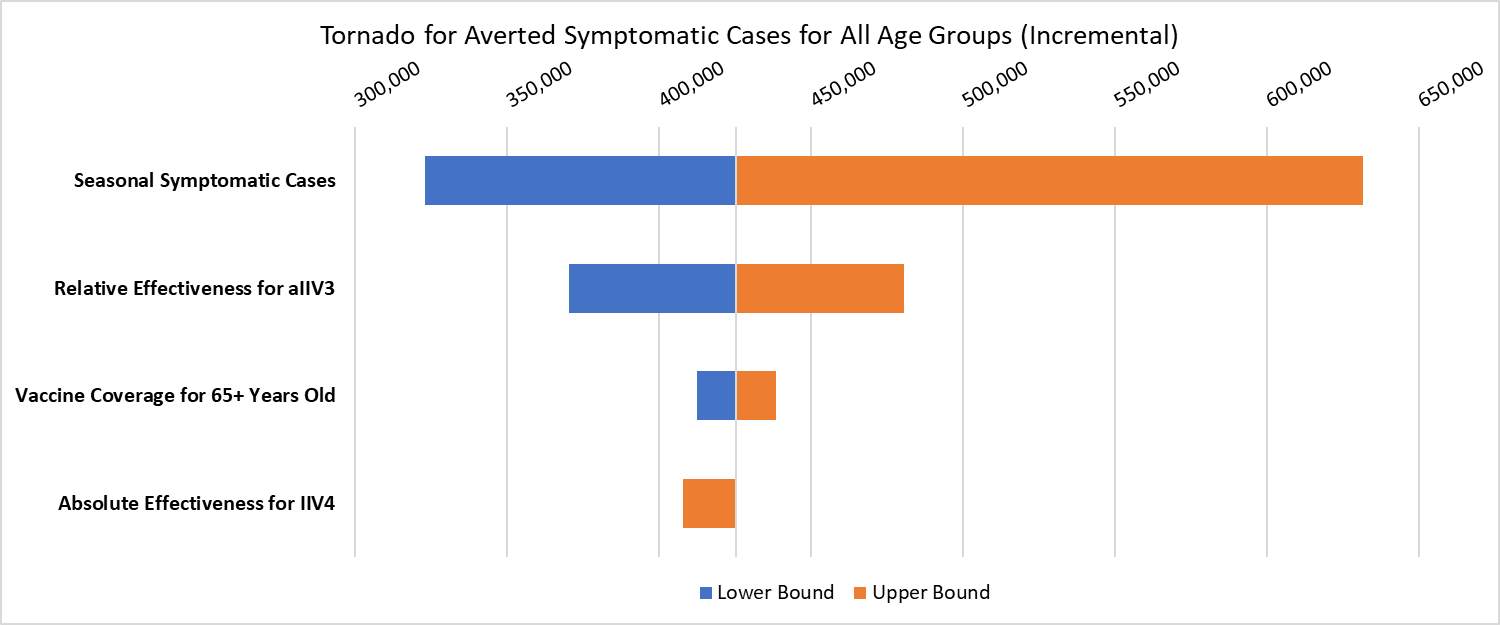
**

| **Rank** | **Parameter Name** | **Lower Bound: Symptomatic Cases Averted** | **Upper Bound: Symptomatic Cases Averted** | **Lower Bound: Percentage Change From Base Case** | **Upper Bound: Percentage Change From Base Case** |
| --- | --- | --- | --- | --- | --- |
|  | Base Case | 425,240 |  |  |  |
| 1 | Seasonal Symptomatic Cases | 323,023 | 631,583 | -24.0% | 48.5% |
| 2 | Relative Effectiveness for aIIV3 | 370,199 | 480,559 | -12.9% | 13.0% |
| 3 | Vaccine Coverage for 65+ Years Old | 412,387 | 438,432 | -3.0% | 3.1% |
| 4 | Absolute Effectiveness for IIV4 | 409,779 | 407,817 | -3.6% | -4.1% |

**Supplementary Figure 3. Tornado Diagram of Additional (aIIV3 vs IIV4) Outpatient Visits Averted in Adults ≥65 Years for the 2019-2020 Influenza Season.** Abbreviations: aIIV3, MF59-adjuvanted trivalent inactivated influenza vaccine; IIV4, generic quadrivalent inactivated influenza vaccine.


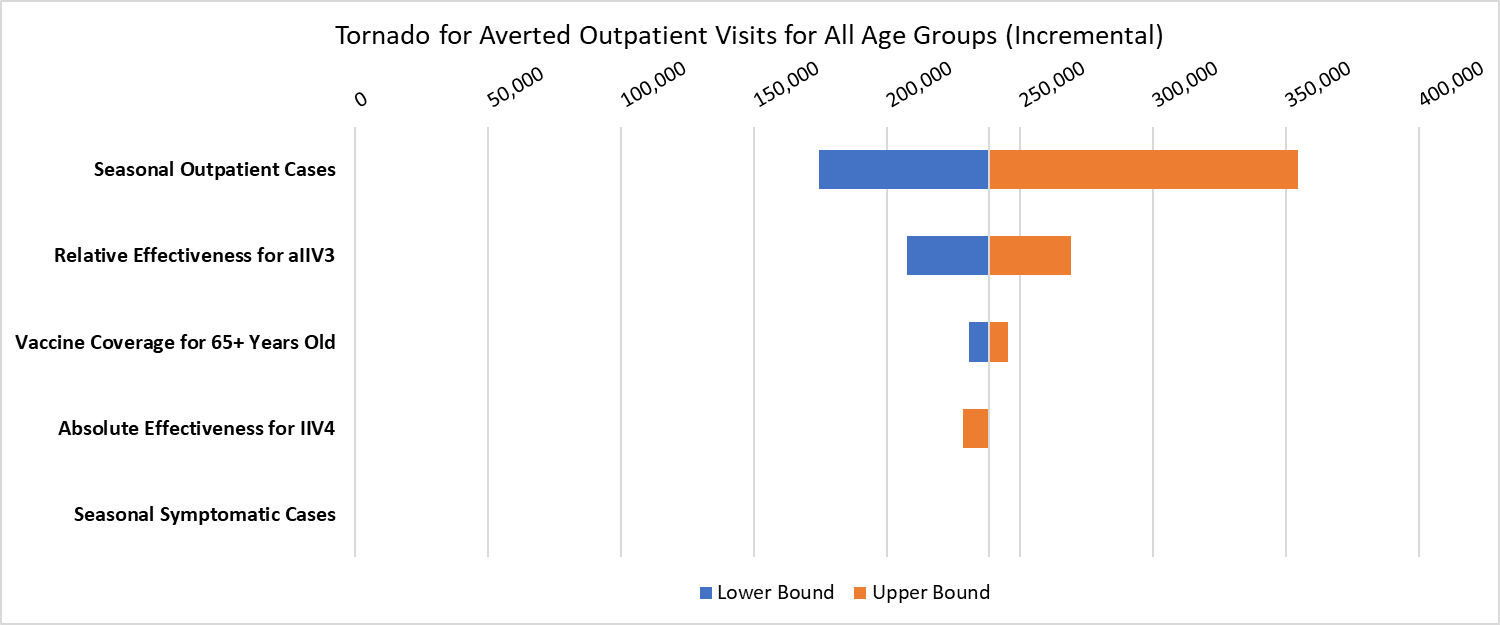


| **Rank** | **Parameter Name** | **Lower Bound: Outpatient Visits Averted** | **Upper Bound: Outpatient Visits Averted** | **Lower Bound: Percentage Change From Base Case** | **Upper Bound: Percentage Change From Base Case** |
| --- | --- | --- | --- | --- | --- |
|  | Base Case | 238,134 |  |  |  |
| 1 | Seasonal Outpatient Cases | 174,353 | 354,353 | -26.8% | 48.8% |
| 2 | Relative Effectiveness for aIIV3 | 207,312 | 269,113 | -12.9% | 13.0% |
| 3 | Vaccine Coverage for 65+ Years Old | 230,936 | 245,522 | -3.0% | 3.1% |
| 4 | Absolute Effectiveness for IIV4 | 229,476 | 228,378 | -3.6% | -4.1% |
| 5 | Seasonal Symptomatic Cases | 237,857 | 238,700 | -0.1% | 0.2% |

**Supplementary Figure 4. Tornado Diagram of Additional (aIIV3 vs IIV4) Hospitalizations Averted in Adults ≥65 Years for the 2019-2020 Influenza Season.** Abbreviations: aIIV3, MF59-adjuvanted trivalent inactivated influenza vaccine; IIV4, generic quadrivalent inactivated influenza vaccine.

**
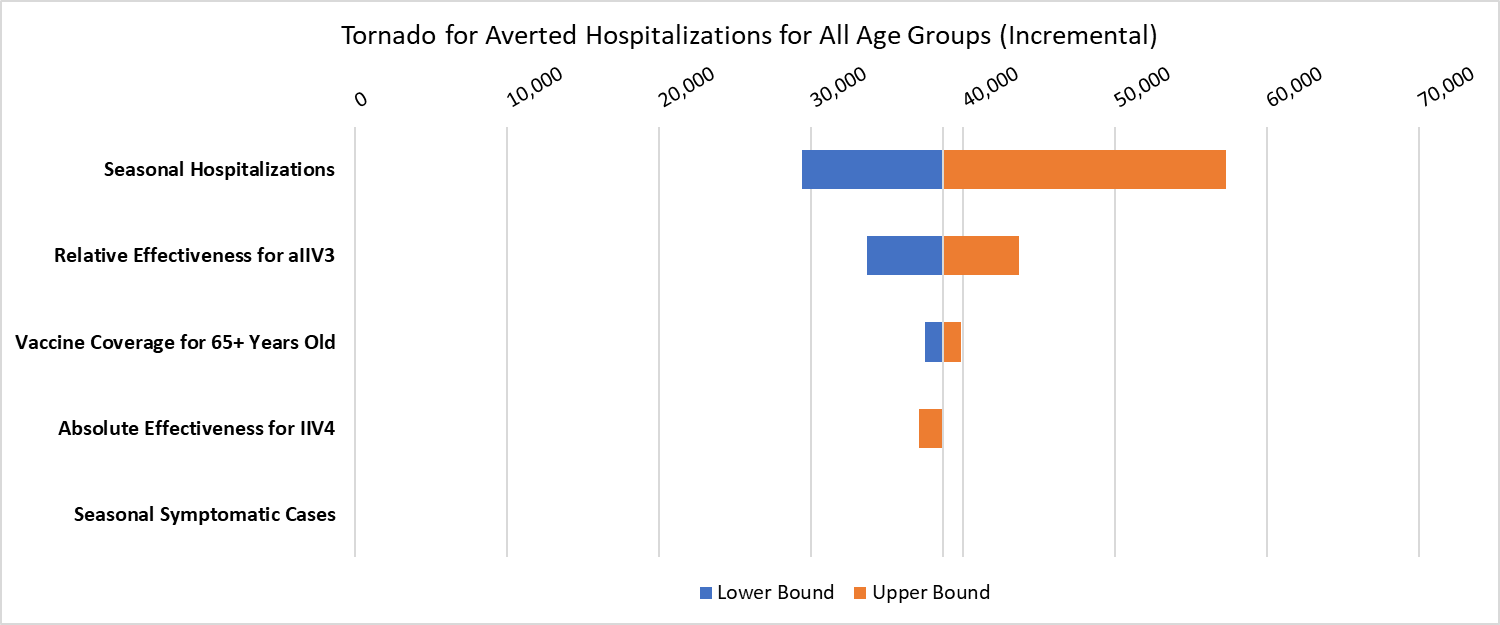
**

| **Rank** | **Parameter Name** | **Lower Bound: Hospitalizations Averted** | **Upper Bound: Hospitalizations Averted** | **Lower Bound: Percentage Change From Base Case** | **Upper Bound: Percentage Change From Base Case** |
| --- | --- | --- | --- | --- | --- |
|  | Base Case | 38,658 |  |  |  |
| 1 | Seasonal Hospitalizations | 29,400 | 57,281 | -23.9% | 48.2% |
| 2 | Relative Effectiveness for aIIV3 | 33,655 | 43,687 | -12.9% | 13.0% |
| 3 | Vaccine Coverage for 65+ Years Old | 37,490 | 39,858 | -3.0% | 3.1% |
| 4 | Absolute Effectiveness for IIV4 | 37,253 | 37,074 | -3.6% | -4.1% |
| 5 | Seasonal Symptomatic Cases | 38,613 | 38,750 | -0.1% | 0.2% |

**Supplementary Figure 5. Tornado Diagram of Additional (aIIV3 vs IIV4) ICU Visits Averted in Adults ≥65 Years for the 2019-2020 Influenza Season.** Abbreviations: aIIV3, MF59-adjuvanted trivalent inactivated influenza vaccine; IIV4, generic quadrivalent inactivated influenza vaccine.

**
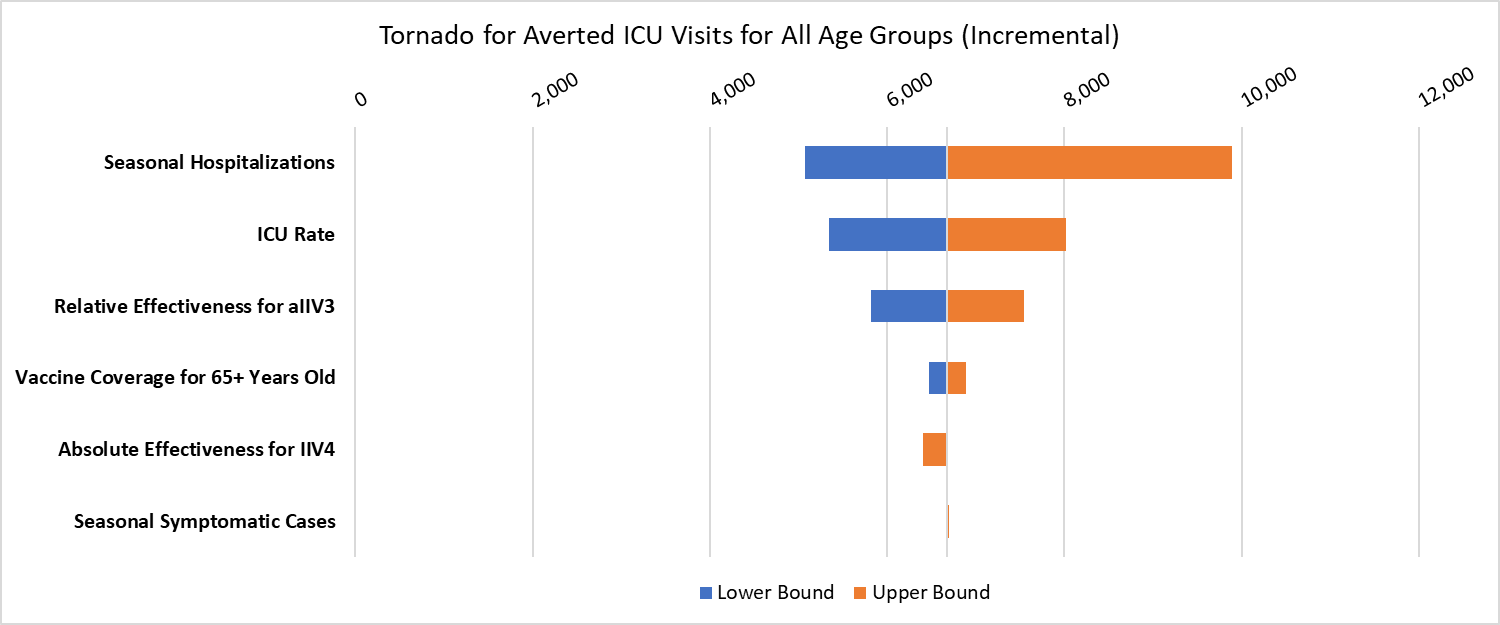
**

| **Rank** | **Parameter Name** | **Lower Bound: ICU Visits Averted** | **Upper Bound: ICU Visits Averted** | **Lower Bound: Percentage Change From Base Case** | **Upper Bound: Percentage Change From Base Case** |
| --- | --- | --- | --- | --- | --- |
|  | Base Case | 6,676 |  |  |  |
| 1 | Seasonal Hospitalizations | 5,077 | 9,892 | -23.9% | 48.2% |
| 2 | ICU Rate | 5,341 | 8,012 | -20.0% | 20.0% |
| 3 | Relative Effectiveness for aIIV3 | 5,812 | 7,545 | -12.9% | 13.0% |
| 4 | Vaccine Coverage for 65+ Years Old | 6,474 | 6,883 | -3.0% | 3.1% |
| 5 | Absolute Effectiveness for IIV4 | 6,434 | 6,403 | -3.6% | -4.1% |
| 6 | Seasonal Symptomatic Cases | 6,669 | 6,692 | -0.1% | 0.2% |

**Supplementary Figure 6. Tornado Diagram of Additional (aIIV3 vs IIV4) Deaths Averted in Adults ≥65 Years for the 2019-2020 Influenza Season.** Abbreviations: aIIV3, MF59-adjuvanted trivalent inactivated influenza vaccine; IIV4, generic quadrivalent inactivated influenza vaccine.

**
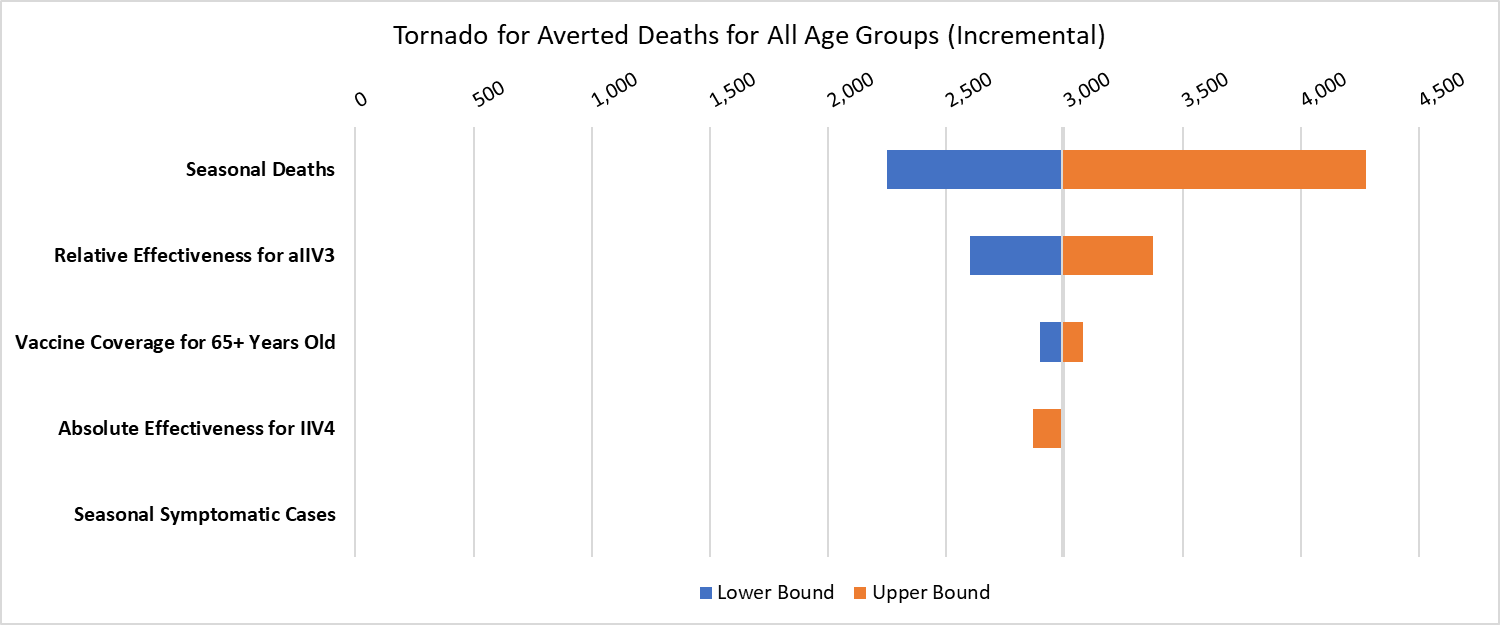
**

| **Rank** | **Parameter Name** | **Lower Bound: Deaths Averted** | **Upper Bound: Deaths Averted** | **Lower Bound: Percentage Change From Base Case** | **Upper Bound: Percentage Change From Base Case** |
| --- | --- | --- | --- | --- | --- |
|  | Base Case | 2,988 |  |  |  |
| 1 | Seasonal Deaths | 2,250 | 4,276 | -24.7% | 43.1% |
| 2 | Relative Effectiveness for aIIV3 | 2,601 | 3,376 | -12.9% | 13.0% |
| 3 | Vaccine Coverage for 65+ Years Old | 2,897 | 3,080 | -3.0% | 3.1% |
| 4 | Absolute Effectiveness for IIV4 | 2,879 | 2,865 | -3.6% | -4.1% |
| 5 | Seasonal Symptomatic Cases | 2,984 | 2,995 | -0.1% | 0.2% |

**Supplementary Figure 7. Tornado Diagram of Additional (aIIV3 vs IIV4) Deaths Averted in Adults ≥65 Years for the 2017-2018 Influenza Season.** Abbreviations: aIIV3, MF59-adjuvanted trivalent inactivated influenza vaccine; IIV4, generic quadrivalent inactivated influenza vaccine.


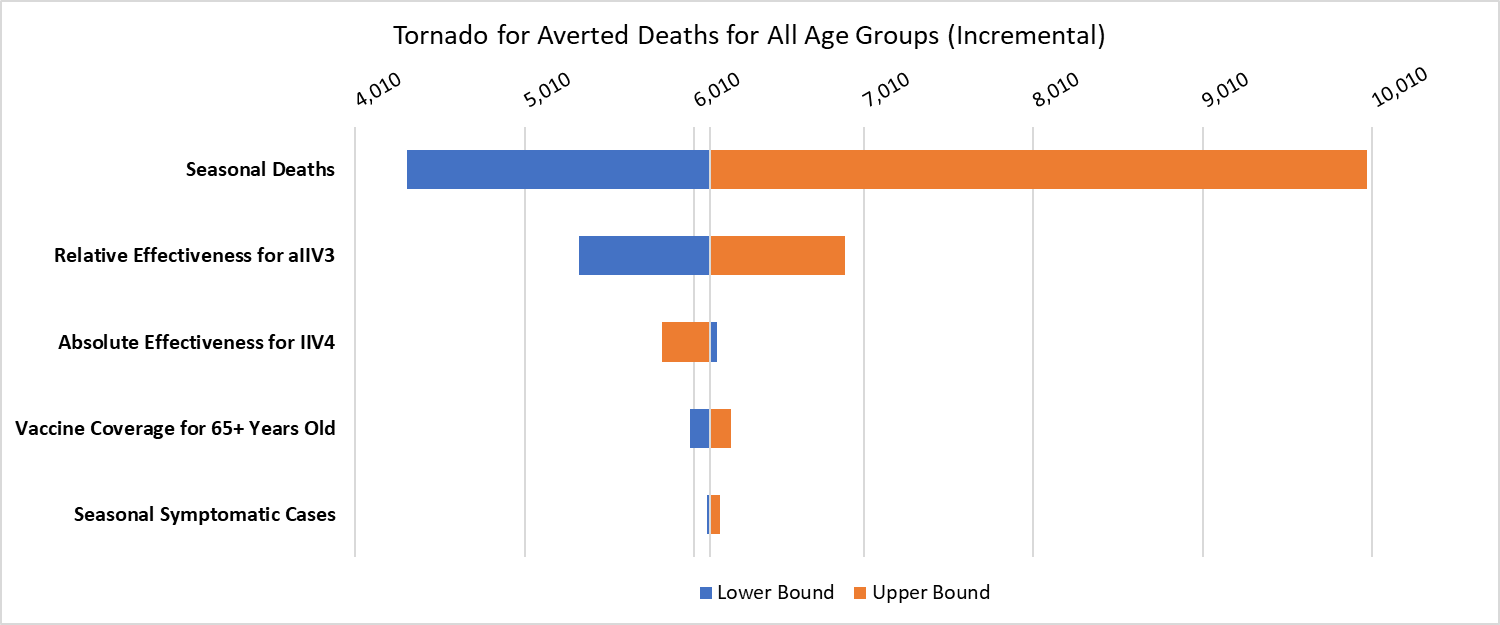


| **Rank** | **Parameter Name** | **Lower Bound: Deaths Averted** | **Upper Bound: Deaths Averted** | **Lower Bound: Percentage Change From Base Case** | **Upper Bound: Percentage Change From Base Case** |
| --- | --- | --- | --- | --- | --- |
|  | Base Case | 6,104 |  |  |  |
| 1 | Seasonal Deaths | 4,316 | 9,980 | -29.3% | 63.5% |
| 2 | Relative Effectiveness for aIIV3 | 5,330 | 6,898 | -12.7% | 13.0% |
| 3 | Absolute Effectiveness for IIV4 | 6,147 | 5,820 | 0.7% | -4.7% |
| 4 | Vaccine Coverage for 65+ Years Old | 5,983 | 6,226 | -2.0% | 2.0% |
| 5 | Seasonal Symptomatic Cases | 6,086 | 6,159 | -0.3% | 0.9% |

**Supplementary Figure 8. Tornado Diagram of Additional (aIIV3 vs IIV4) Symptomatic Cases Averted in Adults ≥65 Years for the 2018-2019 Influenza Season.** Abbreviations: aIIV3, MF59-adjuvanted trivalent inactivated influenza vaccine; IIV4, generic quadrivalent inactivated influenza vaccine.

**
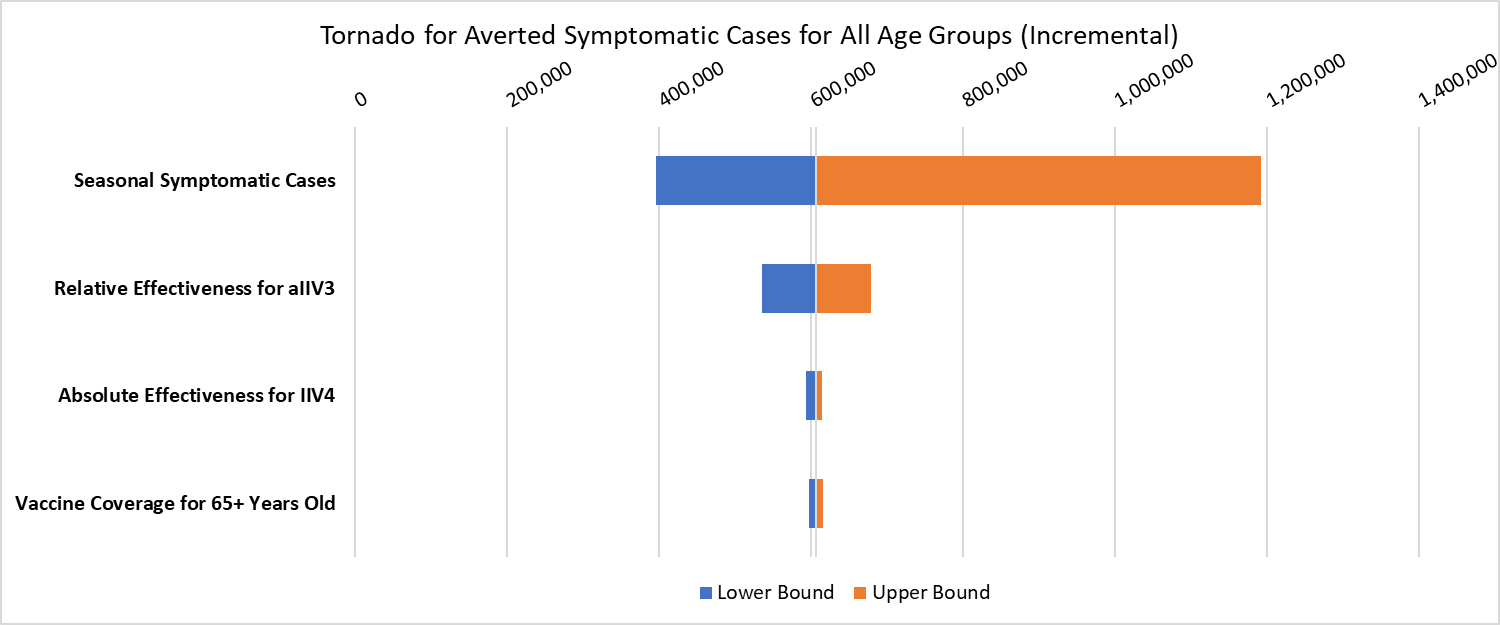
**

| **Rank** | **Parameter Name** | **Lower Bound: Symptomatic Cases Averted** | **Upper Bound: Symptomatic Cases Averted** | **Lower Bound: Percentage Change From Base Case** | **Upper Bound: Percentage Change From Base Case** |
| --- | --- | --- | --- | --- | --- |
|  | Base Case | 605,739 |  |  |  |
| 1 | Seasonal Symptomatic Cases | 395,541 | 1,192,258 | -34.7% | 96.8% |
| 2 | Relative Effectiveness for aIIV3 | 535,239 | 678,908 | -11.6% | 12.1% |
| 3 | Absolute Effectiveness for IIV4 | 593,544 | 614,417 | -2.0% | 1.4% |
| 4 | Vaccine Coverage for 65+ Years Old | 596,639 | 614,912 | -1.5% | 1.5% |

**Supplementary Figure 9. Tornado Diagram of Additional (aIIV3 vs IIV4) Deaths Averted in Adults ≥65 Years for the 2018-2019 Influenza Season.** Abbreviations: aIIV3, MF59-adjuvanted trivalent inactivated influenza vaccine; IIV4, generic quadrivalent inactivated influenza vaccine.

**
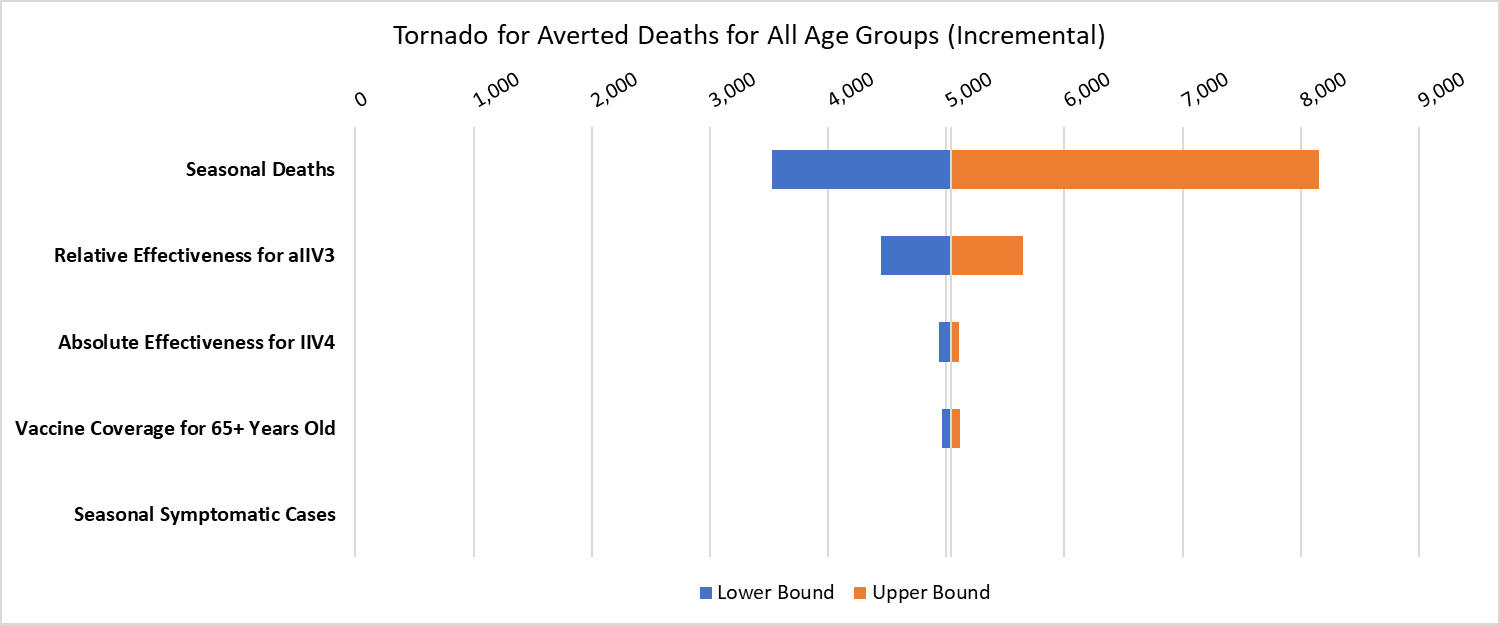
**

| **Rank** | **Parameter Name** | **Lower Bound: Deaths Averted** | **Upper Bound: Deaths Averted** | **Lower Bound: Percentage Change From Base Case** | **Upper Bound: Percentage Change From Base Case** |
| --- | --- | --- | --- | --- | --- |
|  | Base Case | 5,037 |  |  |  |
| 1 | Seasonal Deaths | 3,523 | 8,153 | -30.1% | 61.9% |
| 2 | Relative Effectiveness for aIIV3 | 4,451 | 5,645 | -11.6% | 12.1% |
| 3 | Absolute Effectiveness for IIV4 | 4,936 | 5,109 | -2.0% | 1.4% |
| 4 | Vaccine Coverage for 65+ Years Old | 4,961 | 5,113 | -1.5% | 1.5% |
| 5 | Seasonal Symptomatic Cases | 5,032 | 5,052 | -0.1% | 0.3% |

**Supplementary Figure 10. Tornado Diagram of Additional (aIIV3 vs IIV4) Symptomatic Cases Averted in Adults ≥65 Years for the 2019-2020 Influenza Season Using Method 3.** Abbreviations: aIIV3, MF59-adjuvanted trivalent inactivated influenza vaccine; IIV4, generic quadrivalent inactivated influenza vaccine.

**
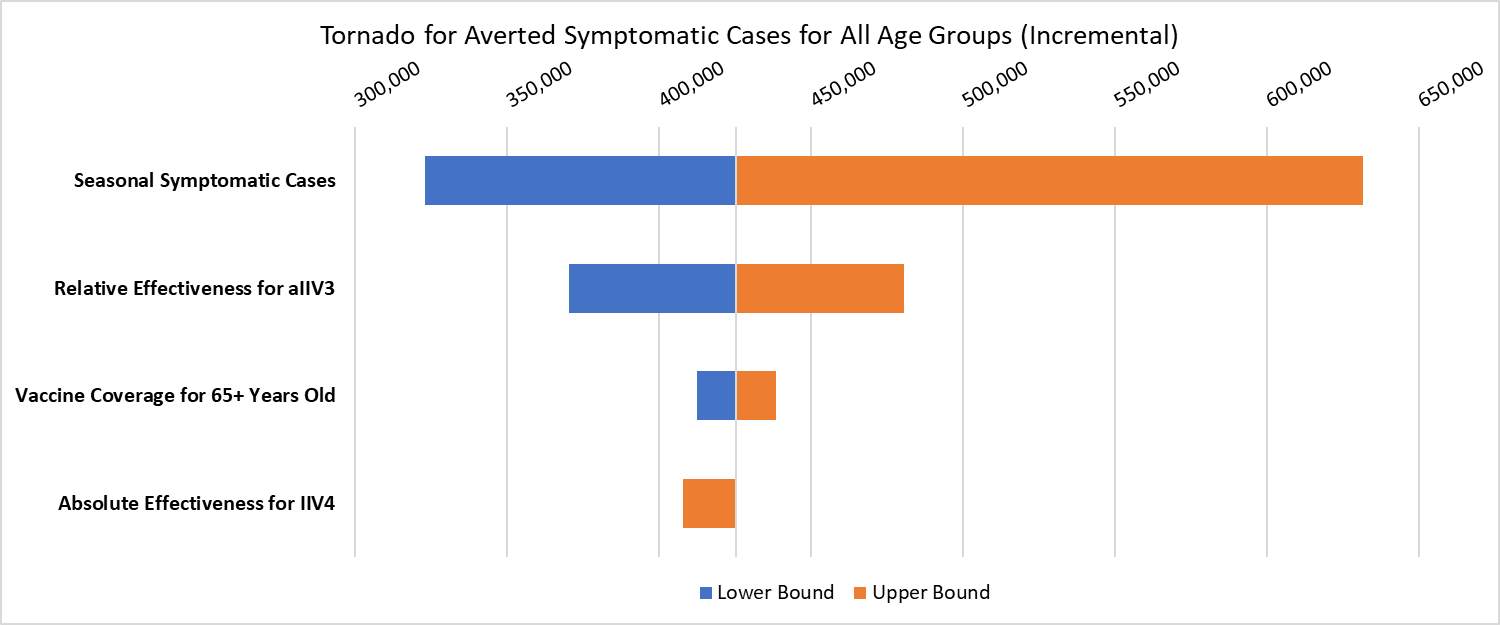
**

| **Rank** | **Parameter Name** | **Lower Bound: Symptomatic Cases Averted** | **Upper Bound: Symptomatic Cases Averted** | **Lower Bound: Percentage Change From Base Case** | **Upper Bound: Percentage Change From Base Case** |
| --- | --- | --- | --- | --- | --- |
|  | Base Case | 425,240 |  |  |  |
| 1 | Seasonal Symptomatic Cases | 323,023 | 631,583 | -24.0% | 48.5% |
| 2 | Relative Effectiveness for aIIV3 | 370,199 | 480,559 | -12.9% | 13.0% |
| 3 | Vaccine Coverage for 65+ Years Old | 412,387 | 438,432 | -3.0% | 3.1% |
| 4 | Absolute Effectiveness for IIV4 | 409,779 | 407,817 | -3.6% | -4.1% |

**Supplementary Figure 11. Tornado Diagram of Additional (aiiv3 vs IIV4) Deaths Averted in Adults ≥65 Years for the 2019-2020 Influenza Season Using Method 3.** Abbreviations: aIIV3, MF59-adjuvanted trivalent inactivated influenza vaccine; IIV4, generic quadrivalent inactivated influenza vaccine.

**
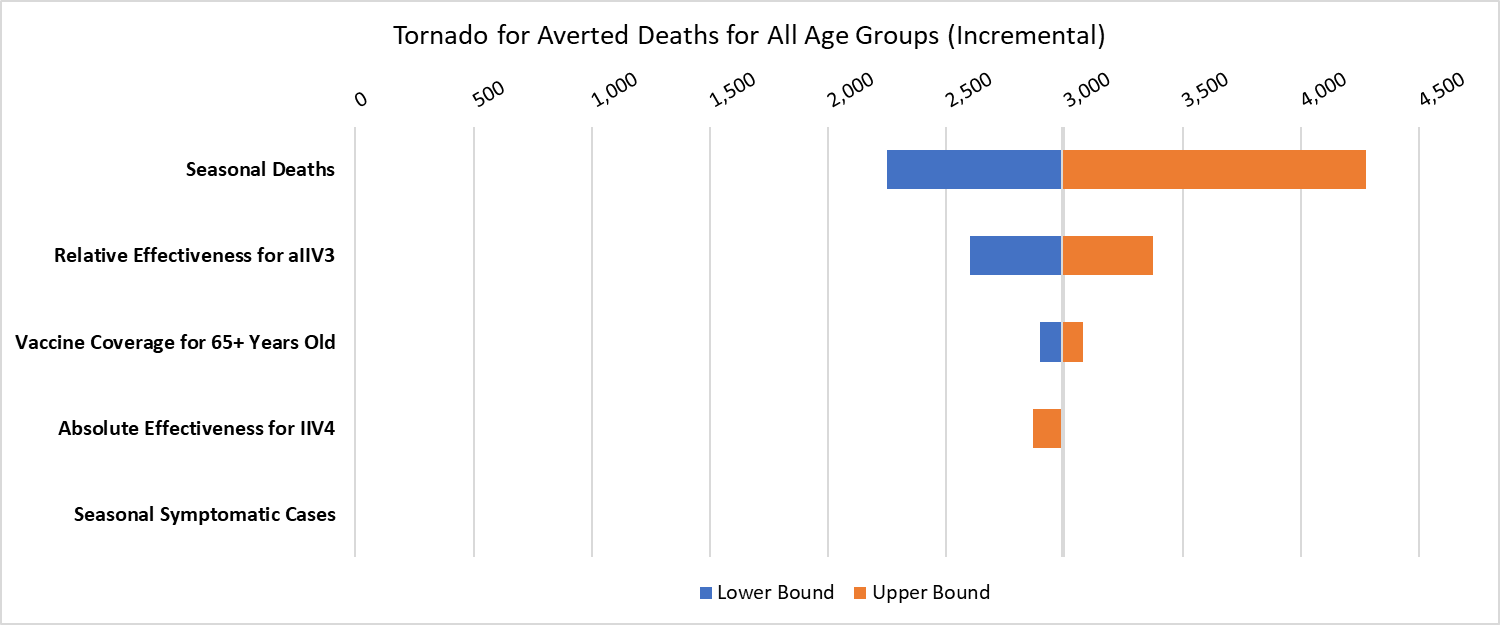
**

| **Rank** | **Parameter Name** | **Lower Bound: Deaths Averted** | **Upper Bound: Deaths Averted** | **Lower Bound: Percentage Change From Base Case** | **Upper Bound: Percentage Change From Base Case** |
| --- | --- | --- | --- | --- | --- |
|  | Base Case | 2,988 |  |  |  |
| 1 | Seasonal Deaths | 2,250 | 4,276 | -24.7% | 43.1% |
| 2 | Relative Effectiveness for aIIV3 | 2,601 | 3,376 | -12.9% | 13.0% |
| 3 | Vaccine Coverage for 65+ Years Old | 2,897 | 3,080 | -3.0% | 3.1% |
| 4 | Absolute Effectiveness for IIV4 | 2,879 | 2,865 | -3.6% | -4.1% |
| 5 | Seasonal Symptomatic Cases | 2,984 | 2,995 | -0.1% | 0.2% |

**Supplementary Figure 12. Distribution of Additional (aIIV3 vs IIV4) Symptomatic Cases Averted in Adults ≥65 Years for the 2017-2018 Influenza Season.** Abbreviations: aIIV3, MF59-adjuvanted trivalent inactivated influenza vaccine; IIV4, generic quadrivalent inactivated influenza vaccine.


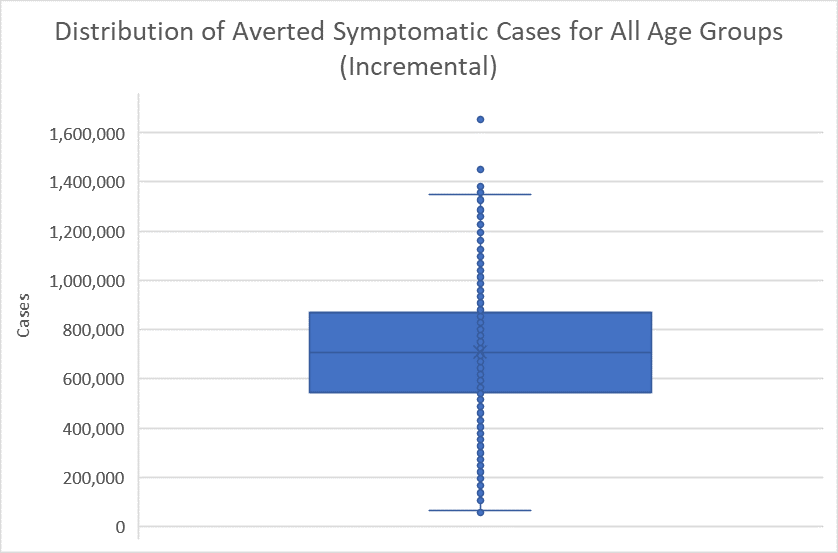


| **Scenario** | **Mean** | **First Quartile** | **Third Quartile** |
| --- | --- | --- | --- |
| aIIV3 | 1,359,408 | 827,503 | 1,698,949 |
| IIV4 | 650,205 | 164,226 | 891,066 |
| Incremental | 709,203 | 545,120 | 868,033 |


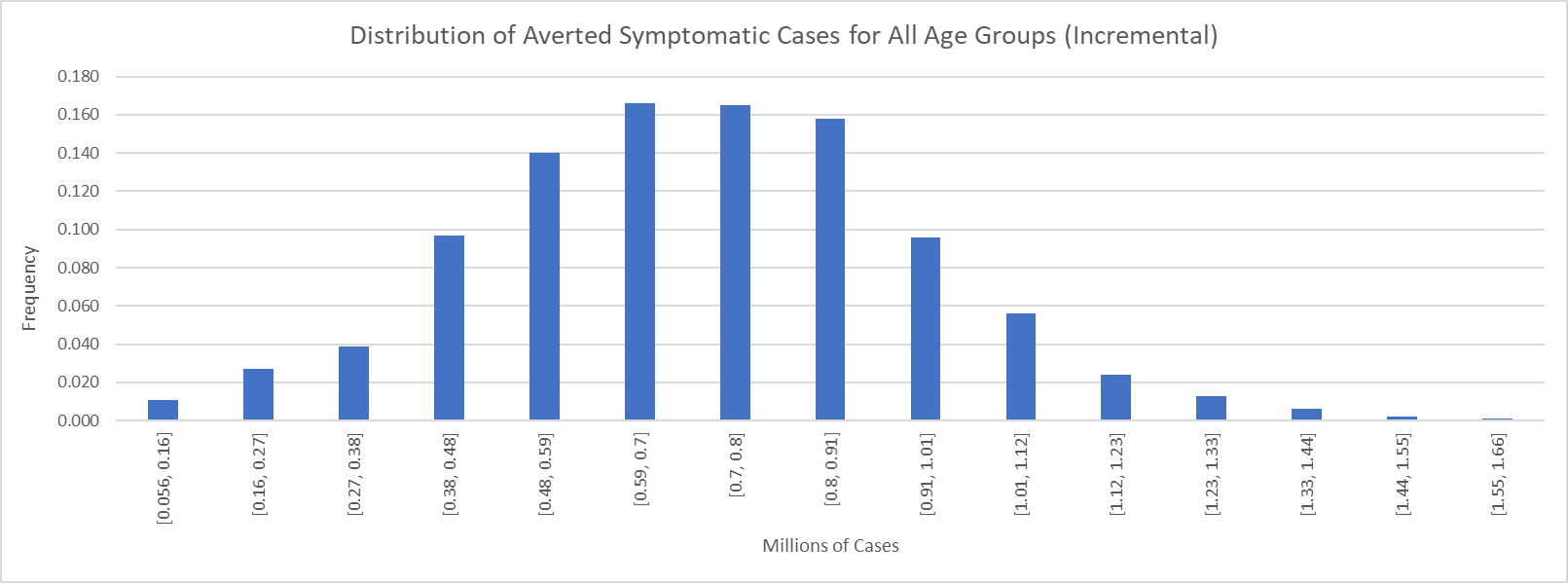


**Supplementary Figure 13. Distribution of Additional (aIIV3 vs IIV4) Deaths Averted in Adults ≥65 Years for the 2017-2018 Influenza Season.** Abbreviations: aIIV3, MF59-adjuvanted trivalent inactivated influenza vaccine; IIV4, generic quadrivalent inactivated influenza vaccine.


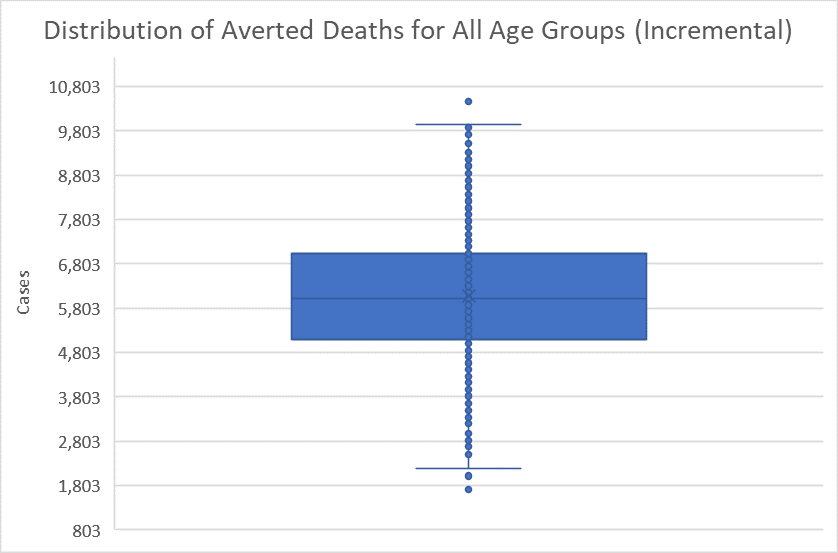


| **Scenario** | **Mean** | **First Quartile** | **Third Quartile** |
| --- | --- | --- | --- |
| aIIV3 | 11,501 | 7,683 | 13,660 |
| IIV4 | 5,424 | 1,684 | 7,440 |
| Incremental | 6,077 | 5,091 | 7,034 |


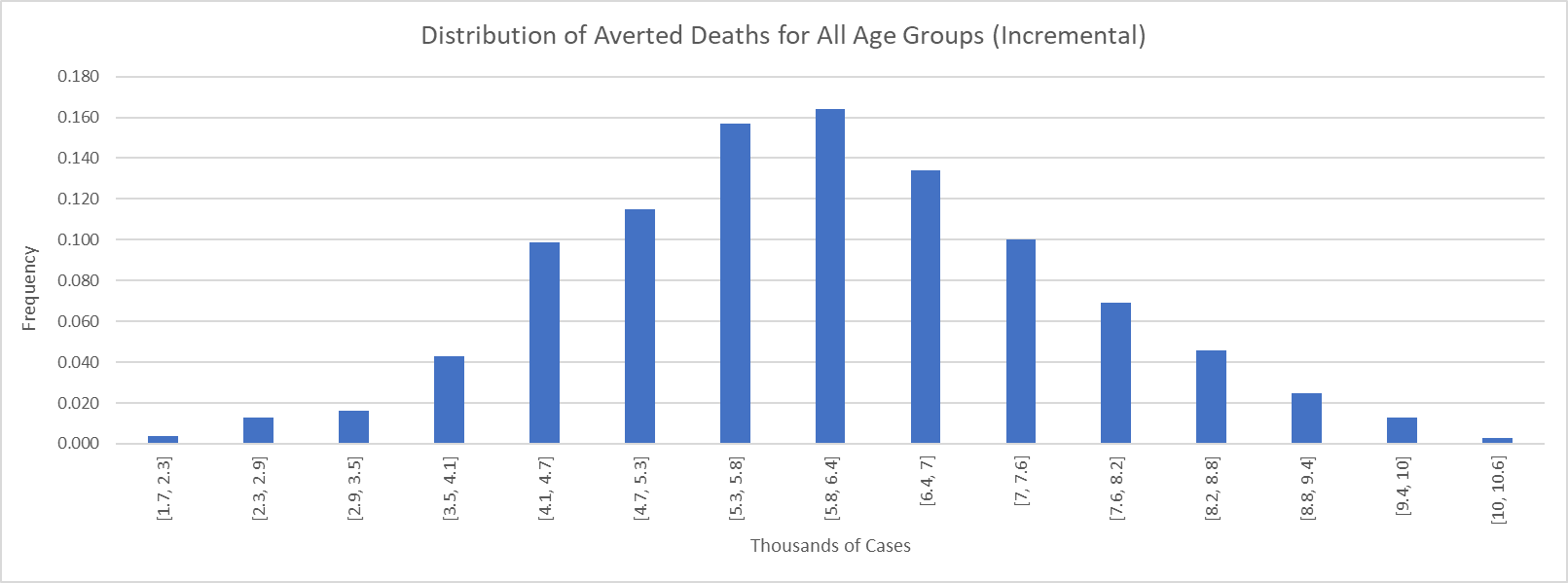


**Supplementary Figure 14. Distribution of Additional (aIIV3 vs IIV4) Symptomatic Cases Averted in Adults ≥65 Years for the 2018-2019 Influenza Season.** Abbreviations: aIIV3, MF59-adjuvanted trivalent inactivated influenza vaccine; IIV4, generic quadrivalent inactivated influenza vaccine.


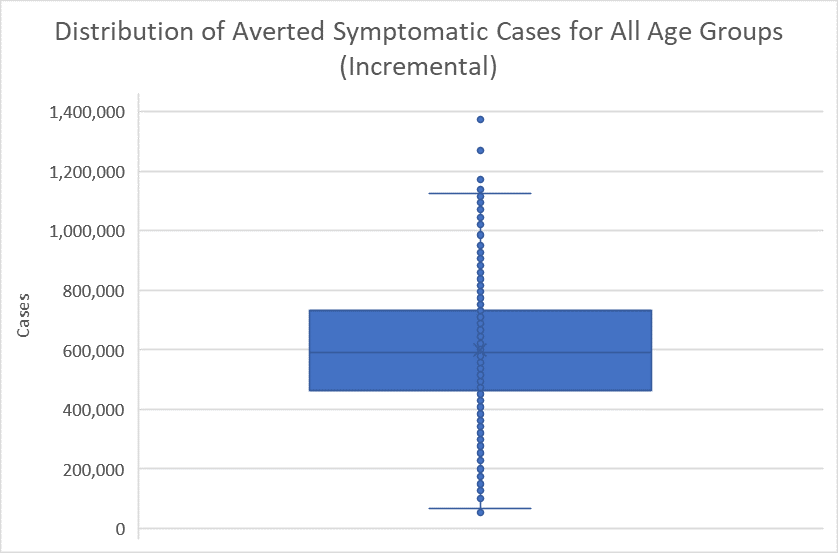


| **Scenario** | **Mean** | **First Quartile** | **Third Quartile** |
| --- | --- | --- | --- |
| aIIV3 | 952,494 | 550,159 | 1,059,514 |
| IIV4 | 352,948 | 3,495 | 373,631 |
| Incremental | 599,546 | 464,593 | 731,501 |


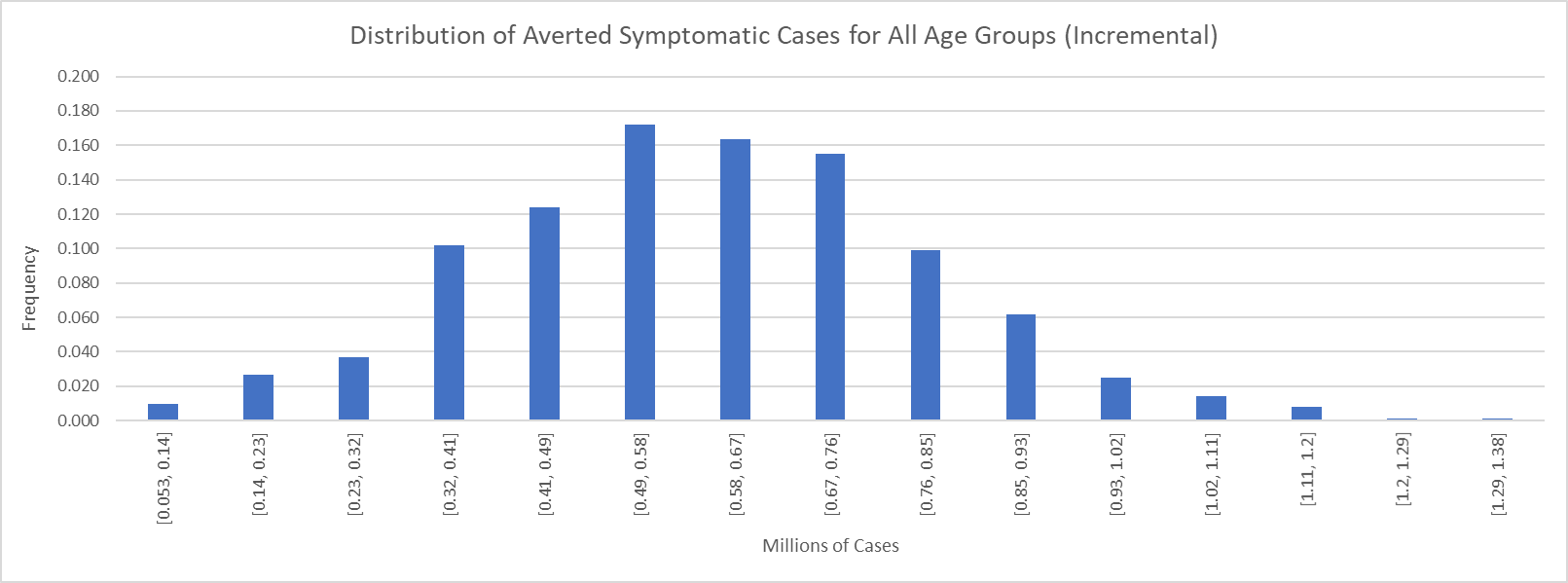


**Supplementary Figure 15. Distribution of Additional (aIIV3 vs IIV4) Deaths Averted in Adults ≥65 Years for the 2018-2019 Influenza Season.** Abbreviations: aIIV3, MF59-adjuvanted trivalent inactivated influenza vaccine; IIV4, generic quadrivalent inactivated influenza vaccine.


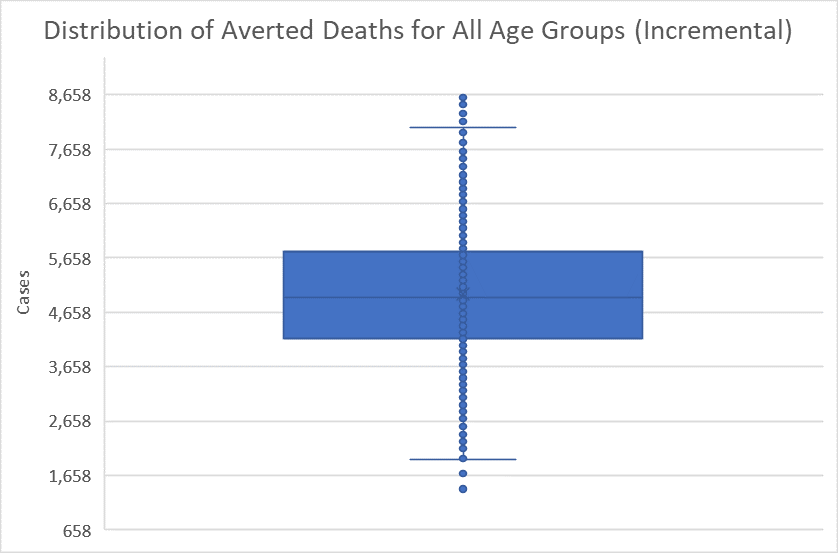


| **Scenario** | **Mean** | **First Quartile** | **Third Quartile** |
| --- | --- | --- | --- |
| aIIV3 | 7,812 | 4,905 | 8,376 |
| IIV4 | 2,823 | 33 | 3,207 |
| Incremental | 4,989 | 4,176 | 5,764 |


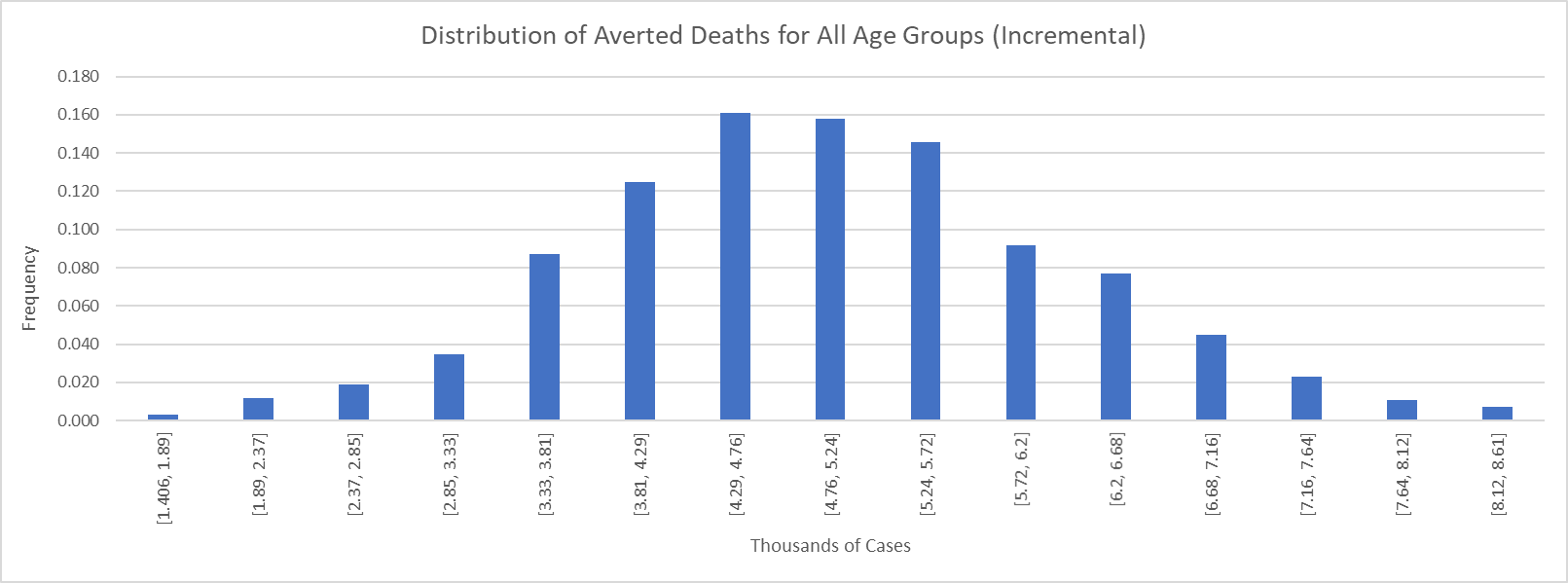


**Supplementary Figure 16. Distribution of Additional (aIIV3 vs IIV4) Symptomatic Cases Averted in Adults ≥65 Years for the 2019-2020 Influenza Season.** Abbreviations: aIIV3, MF59-adjuvanted trivalent inactivated influenza vaccine; IIV4, generic quadrivalent inactivated influenza vaccine.


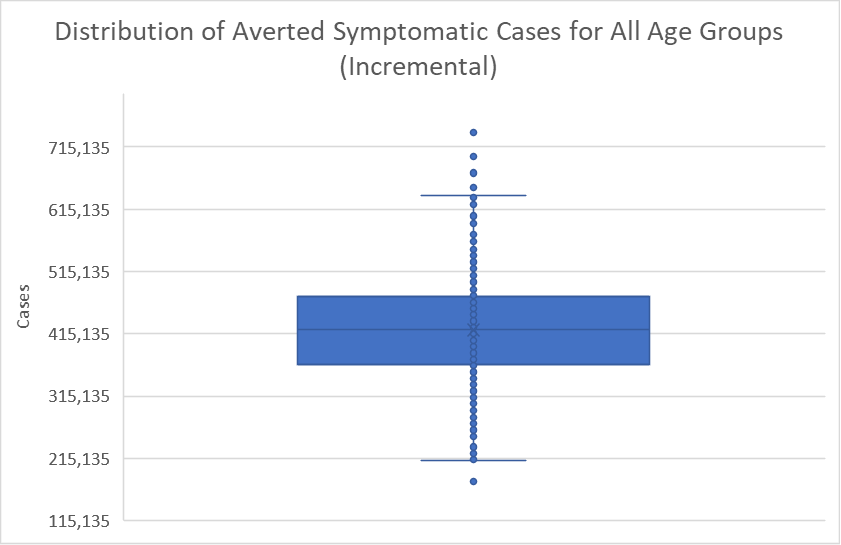


| **Scenario** | **Mean** | **First Quartile** | **Third Quartile** |
| --- | --- | --- | --- |
| aIIV3 | 1,058,349 | 777,771 | 1,278,908 |
| IIV4 | 637,447 | 366,118 | 827,282 |
| Incremental | 420,902 | 365,542 | 474,399 |


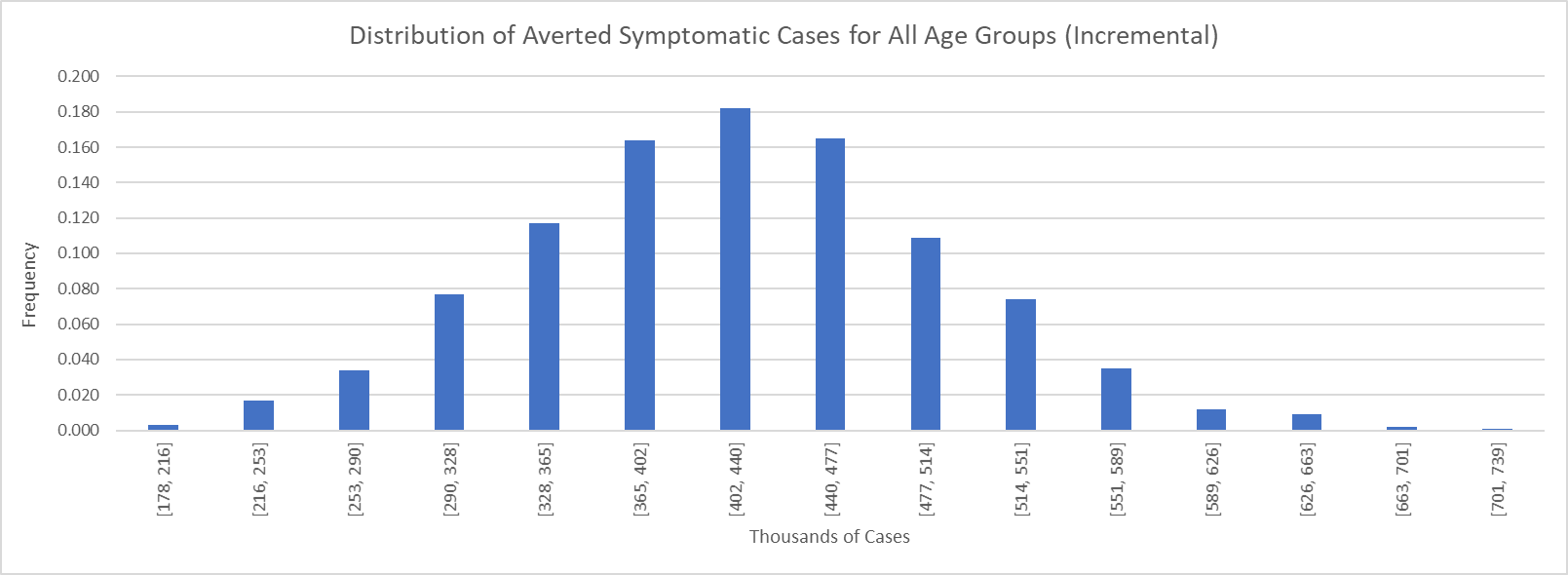


**Supplementary Figure 17. Distribution of Additional (aIIV3 vs IIV4) Outpatient Visits Averted in Adults ≥65 Years for the 2019-2020 Influenza Season.** Abbreviations: aIIV3, MF59-adjuvanted trivalent inactivated influenza vaccine; IIV4, generic quadrivalent inactivated influenza vaccine.


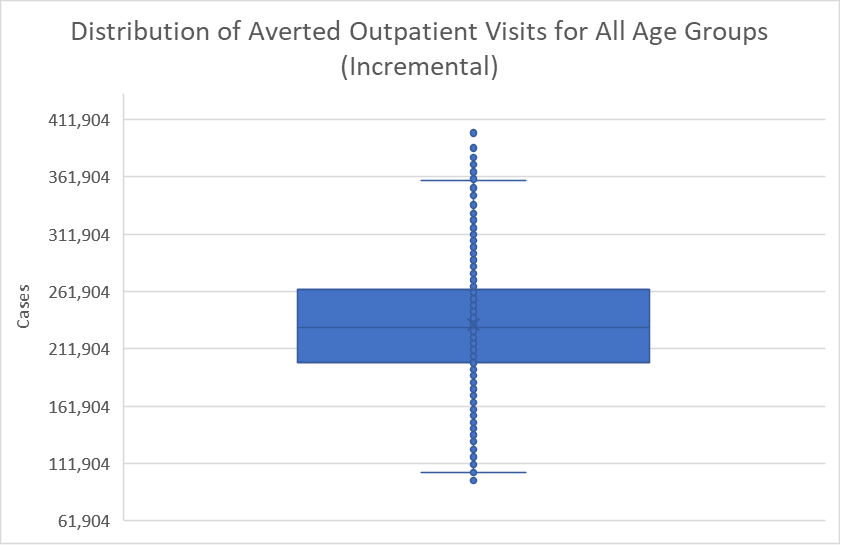


| **Scenario** | **Mean** | **First Quartile** | **Third Quartile** |
| --- | --- | --- | --- |
| aIIV3 | 609,523 | 452,732 | 744,351 |
| IIV4 | 376,909 | 230,438 | 486,474 |
| Incremental | 232,615 | 199,716 | 263,048 |


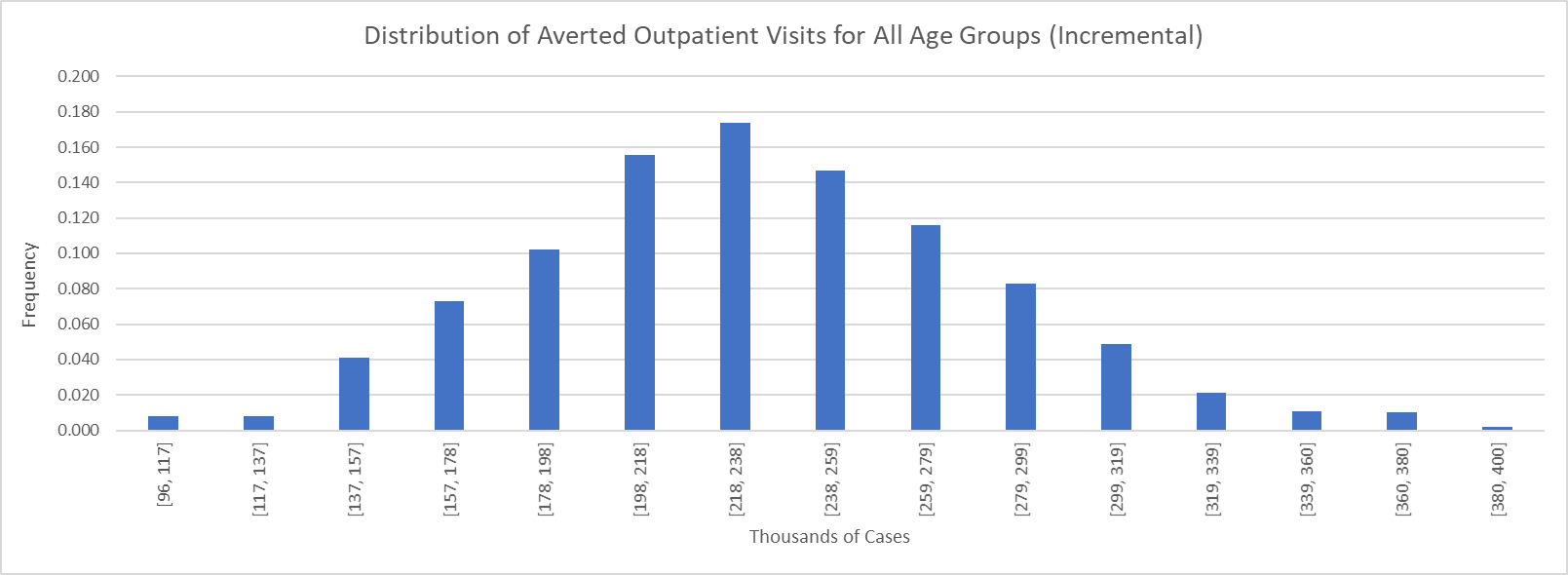


**Supplementary Figure 18. Distribution of Additional (aIIV3 vs IIV4) Hospitalizations Averted in Adults ≥65 Years for the 2019-2020 Influenza Season.** Abbreviations: aIIV3, MF59-adjuvanted trivalent inactivated influenza vaccine; IIV4, generic quadrivalent inactivated influenza vaccine.


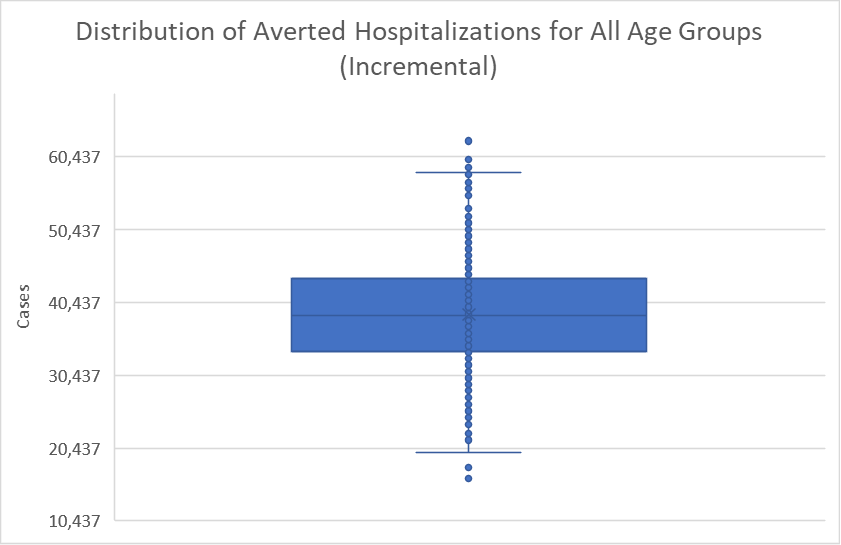


| **Scenario** | **Mean** | **First Quartile** | **Third Quartile** |
| --- | --- | --- | --- |
| aIIV3 | 101,288 | 75,236 | 122,326 |
| IIV4 | 62,577 | 37,973 | 82,455 |
| Incremental | 38,711 | 33,589 | 43,576 |


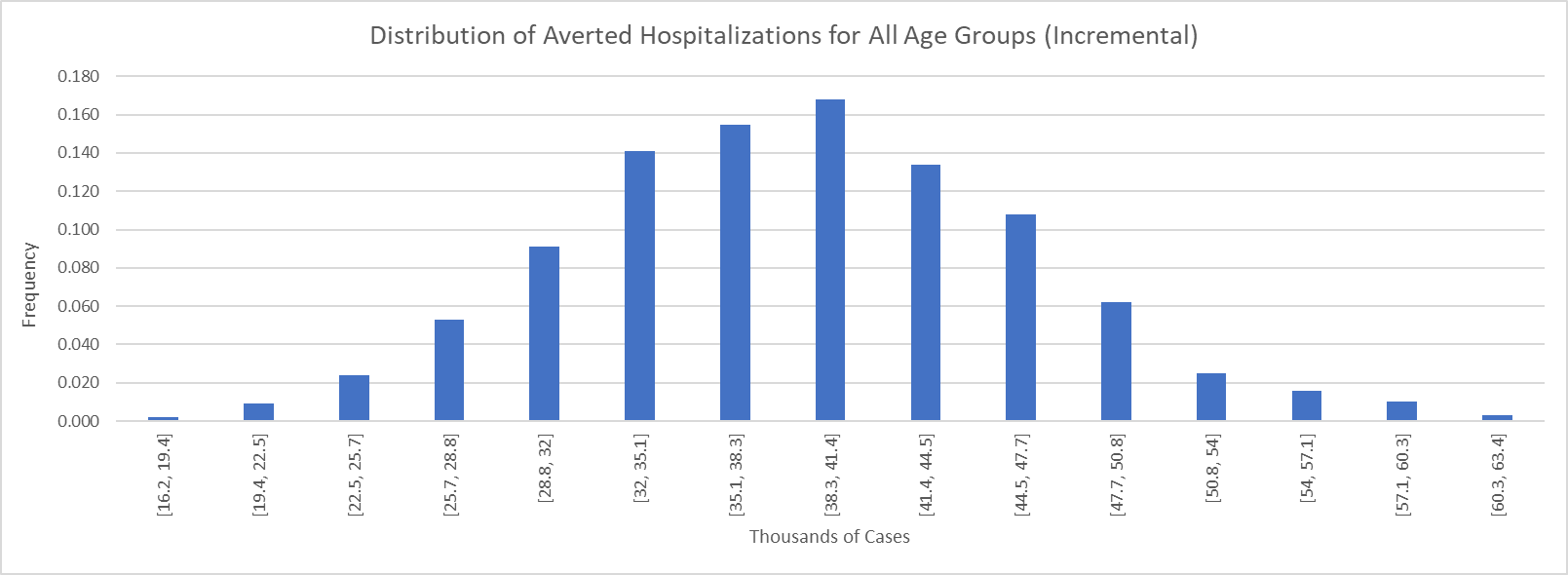


**Supplementary Figure 19. Distribution of Additional (aIIV3 vs IIV4) ICU Visits Averted in Adults ≥65 Years for the 2019-2020 Influenza Season.** Abbreviations: aIIV3, MF59-adjuvanted trivalent inactivated influenza vaccine; IIV4, generic quadrivalent inactivated influenza vaccine.


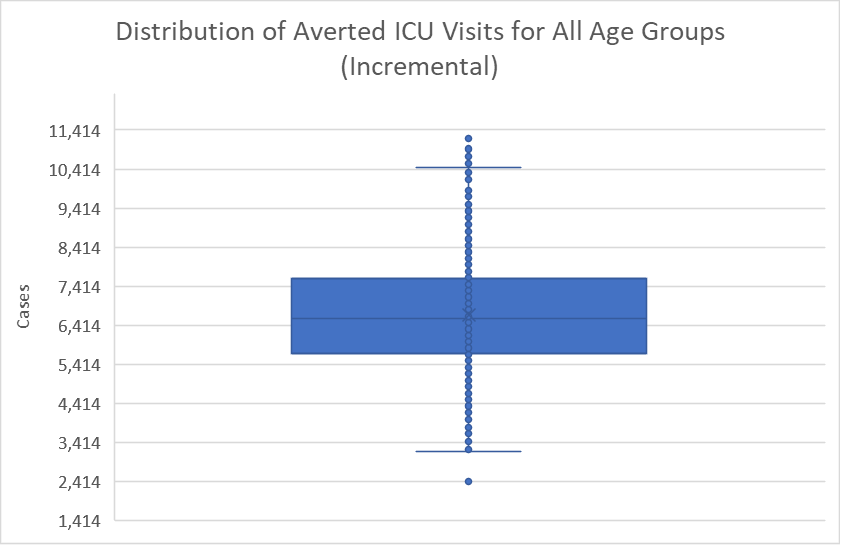


| **Scenario** | **Mean** | **First Quartile** | **Third Quartile** |
| --- | --- | --- | --- |
| aIIV3 | 17,465 | 12,646 | 21,373 |
| IIV4 | 10,797 | 6,351 | 14,418 |
| Incremental | 6,669 | 5,662 | 7,588 |


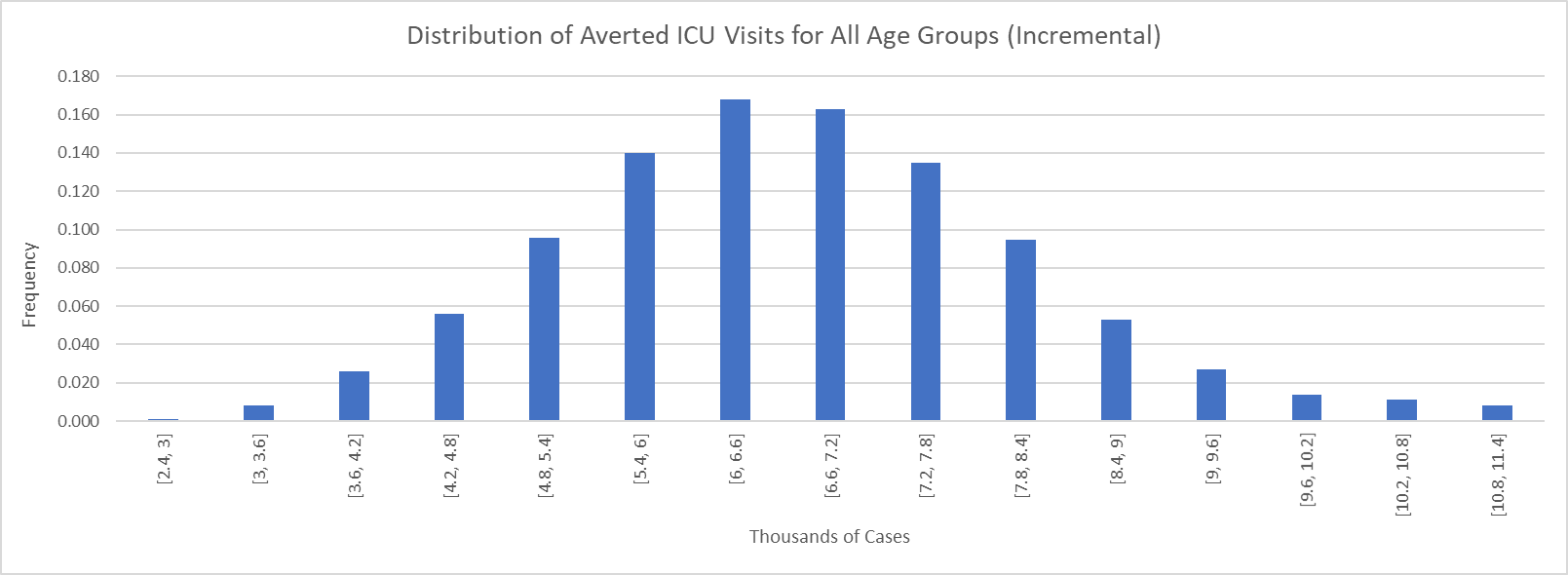


**Supplementary Figure 20. Distribution of Additional (aIIV3 vs IIV4) Deaths Averted in Adults ≥65 Years for the 2019-2020 Influenza Season.** Abbreviations: aIIV3, MF59-adjuvanted trivalent inactivated influenza vaccine; IIV4, generic quadrivalent inactivated influenza vaccine.


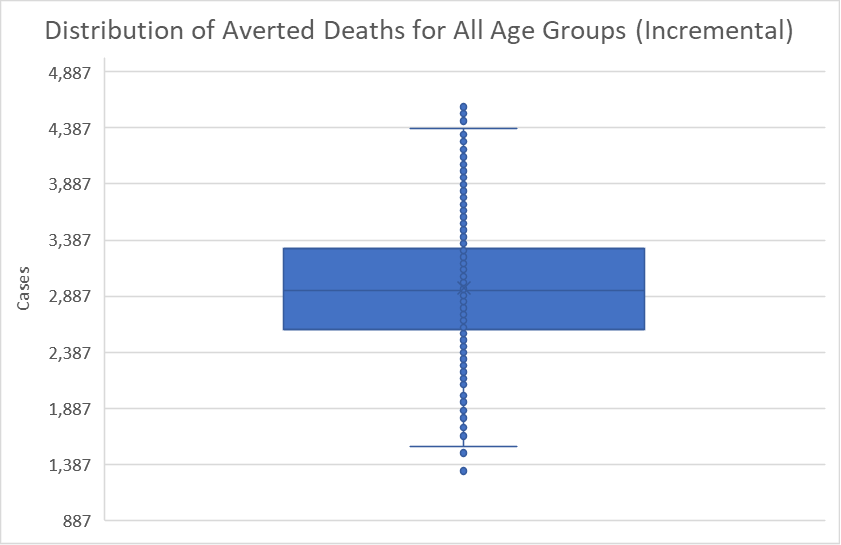


| **Scenario** | **Mean** | **First Quartile** | **Third Quartile** |
| --- | --- | --- | --- |
| aIIV3 | 7,708 | 5,795 | 9,099 |
| IIV4 | 4,752 | 2,987 | 6,042 |
| Incremental | 2,956 | 2,585 | 3,301 |


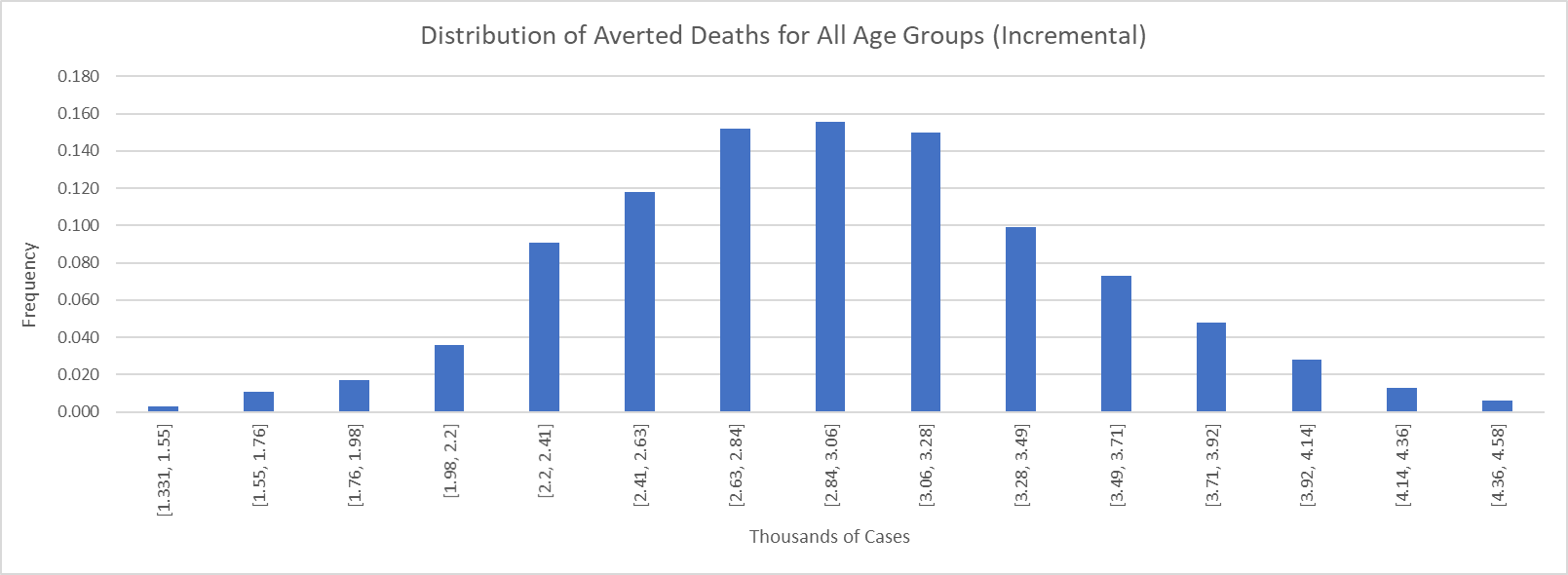


**Supplementary Figure 21. Distribution of Additional (aIIV3 vs IIV4) Symptomatic Cases Averted in Adults ≥65 Years for the 2019-2020 Influenza Season Using Method 3.** Abbreviations: aIIV3, MF59-adjuvanted trivalent inactivated influenza vaccine; IIV4, generic quadrivalent inactivated influenza vaccine.


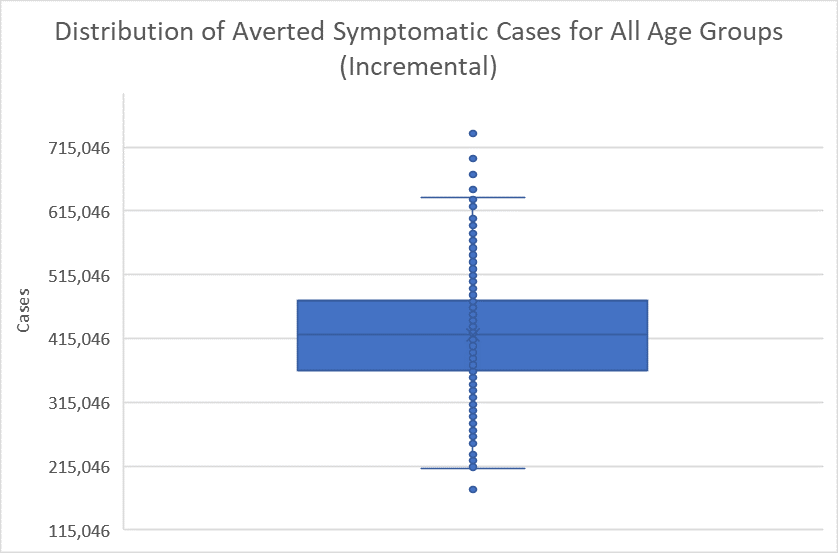


| **Scenario** | **Mean** | **First Quartile** | **Third Quartile** |
| --- | --- | --- | --- |
| aIIV3 | 1,057,468 | 777,323 | 1,277,789 |
| IIV4 | 637,040 | 365,942 | 826,715 |
| Incremental | 420,428 | 365,209 | 437,950 |


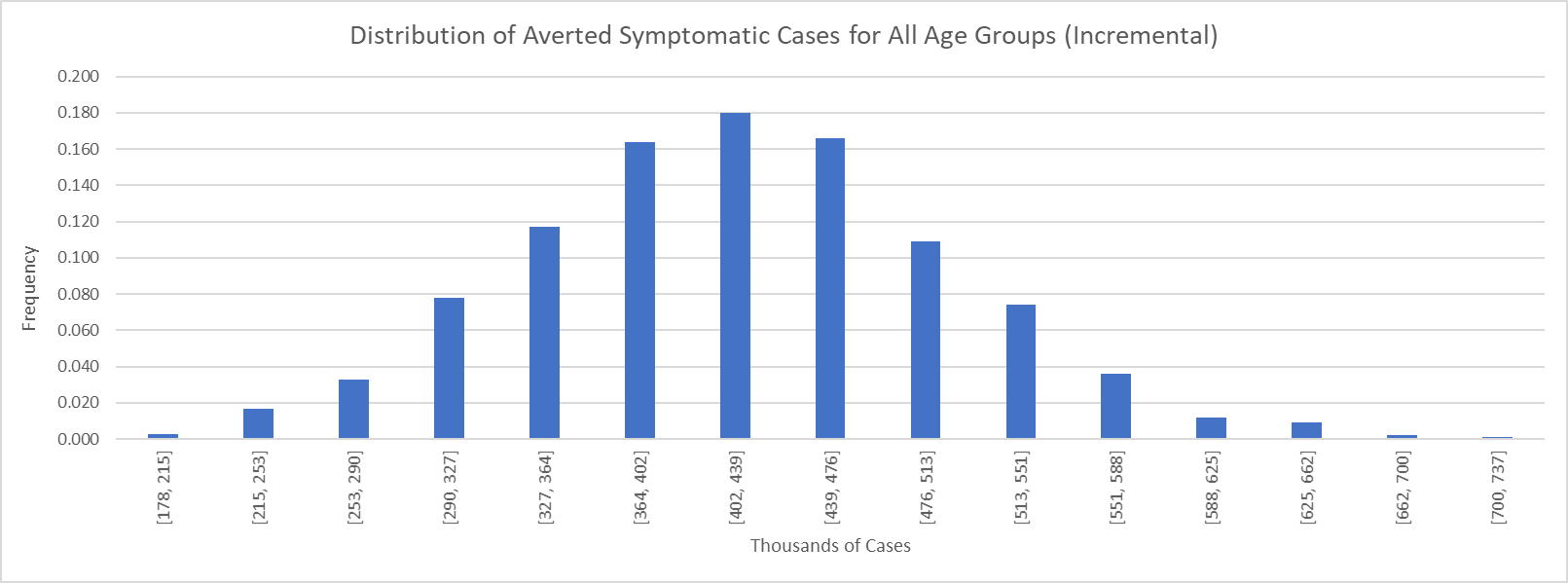


**Supplementary Figure 22. Distribution of Additional (aIIV3 vs IIV4) Deaths Averted in Adults ≥65 Years for the 2019-2020 Influenza Season Using Method 3.** Abbreviations: aIIV3, MF59-adjuvanted trivalent inactivated influenza vaccine; IIV4, generic quadrivalent inactivated influenza vaccine.


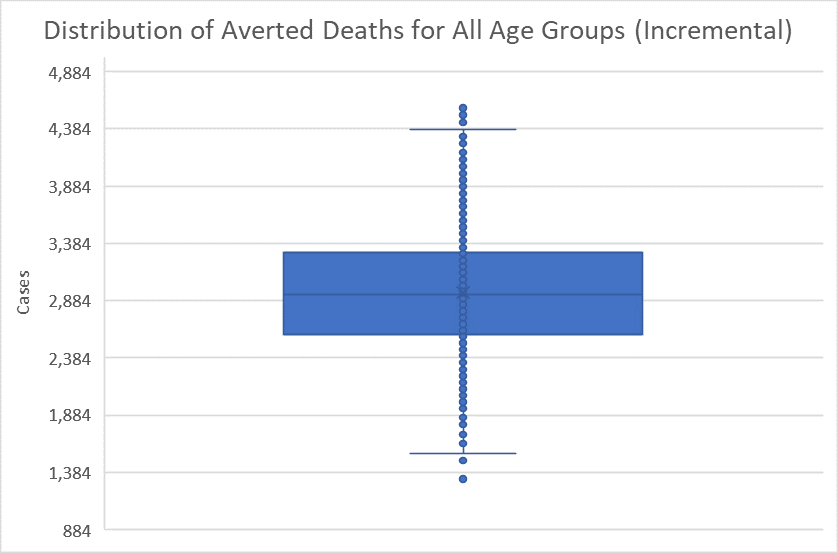


| **Scenario** | **Mean** | **First Quartile** | **Third Quartile** |
| --- | --- | --- | --- |
| aIIV3 | 7,395 | 5,537 | 8,730 |
| IIV4 | 4,437 | 2,716 | 5,689 |
| Incremental | 2,958 | 2,591 | 3,305 |


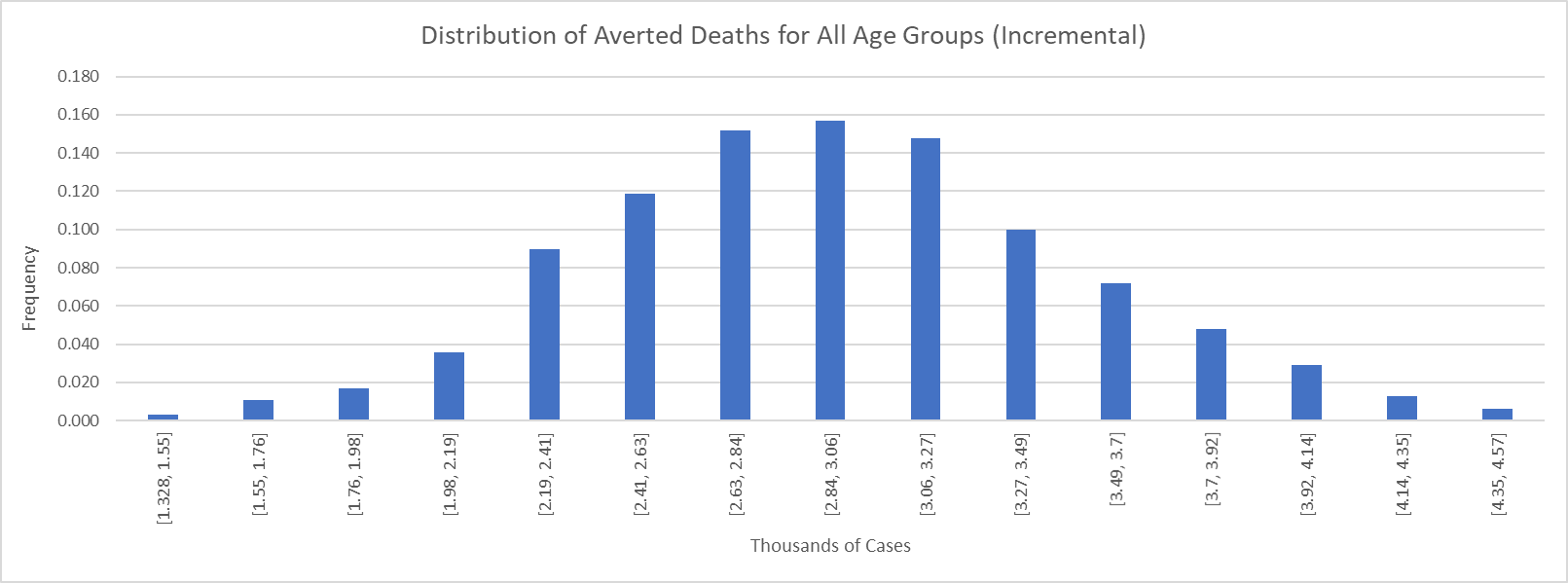

Supplement: ofad429_Supplementary_Data [file ofad429_supplementary_data.docx]
